# Natural history study of hepatic glycogen storage disease type IV and comparison to *Gbe1^ys/ys^* model

**DOI:** 10.1172/jci.insight.177722

**Published:** 2024-05-14

**Authors:** Rebecca L. Koch, Bridget T. Kiely, Su Jin Choi, William R. Jeck, Leticia S. Flores, Vikrant Sood, Seema Alam, Gilda Porta, Katy LaVecchio, Claudia Soler-Alfonso, Priya S. Kishnani

**Affiliations:** 1 Division of Medical Genetics, Department of Pediatrics, and; 2 Department of Pathology, Duke University Medical Center, Durham, North Carolina, USA.; 3 Department of Pediatric Hepatology and Liver Transplantation, Institute of Liver and Biliary Sciences, New Delhi, India.; 4 Hepatology and Liver Transplant Unit, Menino Jesus Hospital, São Paulo, Brazil.; 5 Department of Pathology, The Queen’s Medical Center, Honolulu, Hawaii, USA.; 6 Department of Molecular and Human Genetics, Baylor College of Medicine, Houston, Texas, USA.

**Keywords:** Genetics, Hepatology, Fibrosis, Genetic diseases, Mouse models

## Abstract

**Background:**

Glycogen storage disease type IV (GSD IV) is an ultrarare autosomal recessive disorder that causes deficiency of functional glycogen branching enzyme and formation of abnormally structured glycogen termed polyglucosan. GSD IV has traditionally been categorized based on primary hepatic or neuromuscular involvement, with hepatic GSD IV subclassified as discrete subtypes: classic (progressive) and nonprogressive.

**Methods:**

To better understand the progression of liver disease in GSD IV, we present clinical and histopathology data from 23 patients from around the world and characterized the liver involvement in the *Gbe1^ys/ys^* knockin mouse model.

**Results:**

We propose an alternative to the established subtype-based terminology for characterizing liver disease in GSD IV and recognize 3 tiers of disease severity: (i) “severe progressive” liver disease, (ii) “intermediate progressive” liver disease, and (iii) “attenuated” liver disease. Analysis of liver pathology revealed that risk for liver failure cannot be predicted from liver biopsy findings alone in individuals affected by GSD IV. Moreover, analysis of postmortem liver pathology from an individual who died over 40 years after being diagnosed with nonprogressive hepatic GSD IV in childhood verified that liver fibrosis did not regress. Last, characterization of the liver involvement in a mouse model known to recapitulate the adult-onset neurodegenerative form of GSD IV (*Gbe1^ys/ys^* mouse model) demonstrated hepatic disease.

**Conclusion:**

Our findings challenge the established subtype-based view of GSD IV and suggest that liver disease severity among patients with GSD IV represents a disease continuum.

**Trial registration:**

ClinicalTrials.gov NCT02683512

**Funding:**

None

## Abstract 2

International natural history study of patients with glycogen storage disease type IV and comparison to the *Gbe1^ys/ys^* mouse model characterizes different tiers of liver disease.

## Introduction

Glycogen storage disease type IV (GSD IV; OMIM 232500) is an inborn error of metabolism caused by biallelic pathogenic variants in the *GBE1* gene, which encodes glycogen branching enzyme (GBE), the enzyme responsible for forming branch points during glycogen synthesis. In the setting of deficient GBE activity, the lack of normal branching patterns results in formation of abnormally structured glycogen resembling amylopectin, referred to as polyglucosan, which has long outer glycosidic chains that tangle around one another and form double helices, reducing the solubility of the molecule (1–3). Though GBE is ubiquitously expressed, GSD IV is clinically heterogeneous (4).

In clinical reference materials and published literature, patients have been categorized based on primary hepatic or neuromuscular involvement, with hepatic GSD IV being subclassified as discrete subtypes: (i) classic (progressive) hepatic subtype characterized by rapid development of progressive cirrhosis causing death within first 5 years of life without liver transplantation and (ii) nonprogressive hepatic subtype retrospectively assigned to patients who survive without apparent progression of liver disease as noted in the classic (progressive) hepatic subtype (5). Additional subtypes have been coined to describe individuals with GSD IV and primarily neuromuscular involvement, including the (i) “fatal perinatal neuromuscular” subtype presenting in utero with fetal akinesia deformation sequence, polyhydramnios, and fetal hydrops, progressing to neonatal death; (ii) “congenital/neonatal neuromuscular” subtype presenting in the neonatal period with hypotonia, respiratory distress, and cardiomyopathy, causing death in early infancy; and (iii) “childhood/juvenile neuromuscular” subtype presenting with muscle involvement in the second decade with varying disease severity. On the other end of the phenotypic spectrum is the adult-onset form of GSD IV, referred to as adult polyglucosan body disease (APBD; OMIM 263570), a neurodegenerative disorder not typically associated with overt liver disease (6). Recent work has argued against the utility of the traditional subtype classification system, instead recognizing GSD IV as a multidimensional clinical continuum, whereby hepatic, neurologic, muscular, and cardiac involvement occur to varying degrees in different patients (4).

Historically, the diagnosis of GSD IV with demonstration of fibrosis on liver biopsy immediately called for urgent liver transplantation (LT), largely driven by concern that patients could quickly decompensate and progress to liver failure before a liver was available for transplantation. Recent clinical practice guidelines on the management of GSD IV published in 2023 recommended avoidance of preemptive LT without decompensated cirrhosis because of the possibility of stability or slower progression, lack of available livers, and risk for heart failure after LT (7). The utility of common clinical surveillance methods, including laboratory investigations, liver imaging, and liver biopsy, for predicting risk for liver failure in GSD IV remains unclear. There is a paucity of longitudinal data and comprehensive liver pathology data, leading to variability in management and practice of LT.

Our study represents the largest natural history study of hepatic GSD IV to date, encompassing over 2 decades of follow-up data from 23 patients around the world. Our findings challenge the current paradigm of hepatic GSD IV and suggest that rate of liver disease in GSD IV is variable, with some experiencing rapidly progressive liver disease, while others demonstrate slowly progressive or more of an attenuated liver disease. We conclude that liver failure cannot be predicted from liver biopsy findings alone in individuals with GSD IV. Additionally, given the recent reports of individuals with APBD who had a history of liver disease in childhood (8–10), we characterized the liver involvement in the *Gbe1^ys/ys^* knockin mouse model, which harbors the common pathogenic variant (p.Y329S) observed in APBD (11), improving our understanding of the connection between liver disease in GSD IV and APBD.

## Results

### Patients with hepatic GSD IV exhibit varying rates of liver disease progression and severity

A summary of patient characteristics is included in Table 1. For 21 patients in this study, the diagnosis of GSD IV was based on enzymology studies and/or genetic testing. For the 2 patients without available GBE enzymology studies or *GBE1* genetic testing (patients C8 and C43), the diagnosis of GSD IV was established based on the identification of characteristic histopathological findings on liver biopsy. The full clinical course of each patient is detailed in Supplemental Data 1; supplemental material available online with this article; https://doi.org/10.1172/jci.insight.177722DS1 Sibling pairs included patients C11/C12 and C26/C32. Patients C3, C7, C9, C11, C12, and C45 were previously described in other publications (Table 1), with additional follow-up data reported in the current study.

### LT, survival, and long-term follow-up

**Figure 1:**
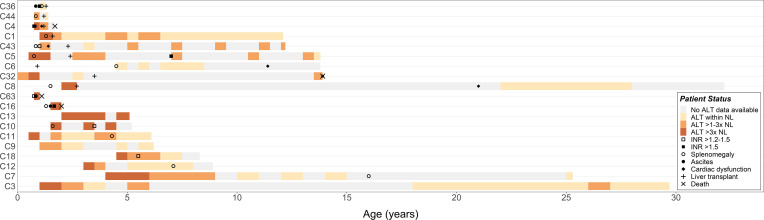
The natural history of hepatic GSD IV reveals a spectrum of liver disease. Longitudinal patient data are shown in order of age at latest follow-up (youngest to oldest) and liver transplantation status (underwent a liver transplant: C36 to C8; did not undergo an LT: C63 to C3). Shaded levels of ALT correspond to one 3-month period (for patients with ≤ 2 years of laboratory follow-up), 6-month period (2–20 years of follow-up), or 12-month period (≥ 20 years of follow-up) starting with the first documented level. Cases without specific ALT levels available were excluded from the plot (C26, C45, C61, C66). First evidence of INR > 1.2–1.5 and INR > 1.5 are indicated, except for C8, who was on anticoagulation therapy at the time of abnormal INR. If ALT and/or INR was measured more than once during a given interval, the values were averaged. First report of splenomegaly, ascites, and cardiac dysfunction by imaging and/or clinical examination are plotted. Full details on each case are in Supplemental Data 1. ALT, alanine aminotransferase; INR, international normalized ratio; NL, normal limits.

Five patients were deceased as of the most recent available follow-up. These included 2 patients who developed liver failure while on the LT waiting list: C16 died of septic shock and disseminated intravascular coagulation at 2.0 years of age, and C63 died of refractory multiorgan failure at 1.1 years of age. Of the 12 patients who underwent LT at a median of 2.0 years of age (range: 0.9 to 3.5 years), C4 died from complications of cardiac dysfunction at 1.7 years of age (6 months after LT), and C32 died because of transplant rejection at 13.9 years of age (10.4 years after LT). Other reported post-LT complications included biliary strictures and cholangitis (C5), acute cellular rejection (C1, C26), persistent hypertension (C26), and posterior reversible encephalopathy syndrome (C26). One patient (C45) previously diagnosed with the nonprogressive hepatic subtype in childhood was diagnosed with APBD at age 38 years after presenting with symptoms of urinary hesitancy, lower limb weakness, and gait disturbance (10). C45 died at the age of 47.8 years; at time of death, the patient did not have overt liver disease. The 18 surviving patients (10 LT, 8 non-LT) were followed through a median age of 12.2 years (range: 1.3 to 32.6 years). Longitudinal disease progression is shown in Figure 1.

### Imaging and liver biopsy findings

**Figure 2:**
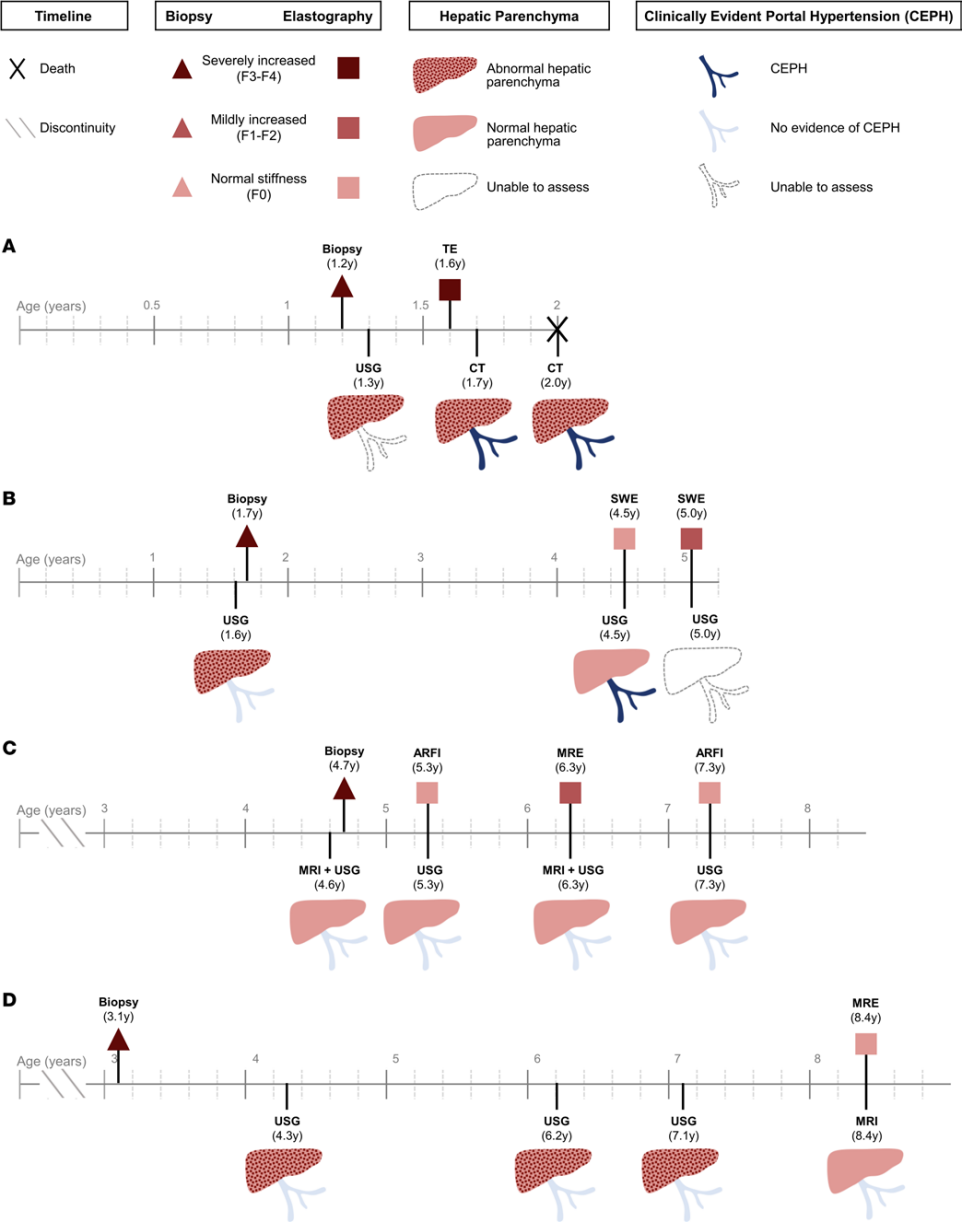
Evaluation of extent of liver fibrosis by elastography, biopsy, and conventional imaging studies in patients with hepatic GSD IV. Findings from liver biopsies, conventional imaging studies (USG, CT, MRI), and elastography studies (TE, SWE, ARFI, MRE) are shown for patients with at least 1 available elastography measurement in order of current age (youngest to oldest). Unless deceased, the ends of timelines indicate age at latest follow-up. Invasive (biopsy) and noninvasive (elastography) assessments of hepatic fibrosis were classified based on a color-coding scheme, with darker shades of red corresponding to greater severity. The degree of fibrosis was based on original pathology reports. Elastography results were coded as demonstrating normal liver stiffness, mildly increased stiffness, or severely increased stiffness. Conventional imaging studies were coded based on radiology reports according to whether there was evidence of hepatic parenchymal abnormalities and CEPH. (**A**) Patient C16: TE at 1.6 years of age showed liver stiffness of 66 kPa, above the cutoff of 11.5 kPa predictive of F4 fibrosis in children. (**B**) Patient C10: Per reference ranges given in the imaging reports, SWV of 1.0 m/s at age 4.5 years was normal, and SWV of 1.65 at age 5.0 years was consistent with F1 fibrosis range. (**C**) Patient C18: ARFI SWV at age 5.3 years (1.25 m/s) and 7.3 years (1.17 m/s) was within normal range based on published reference ranges. MRE at 7.3 years (2.81 kPa) was consistent with mildly elevated stiffness compared with normal range of < 2.75 kPa given in the imaging report. (**D**) Patient C12: MRE at 8.4 years (1.74 kPa) was normal based on reference range of < 2.75 kPa given in the imaging report. USG, ultrasonography; CEPH, clinically evident portal hypertension; CT, computed tomography; MRI, magnetic resonance imaging; MRE, magnetic resonance elastography; SWE, shear wave elastography; SWV, shear wave velocity; ARFI, acoustic radiation force impulse elastography; TE, transient elastography.

Imaging findings and comparison to liver pathology reports are shown for the 4 patients who had at least 1 available hepatic elastography study (C10, C12, C16, C18; Figure 2). All patients with available liver biopsy data (*n* = 19) were reported to exhibit advanced fibrosis or cirrhosis on their initial biopsies at a median of 1.6 years of age (range: 0.3 to 4.6 years) (Table 1, Supplemental Data 1, and Supplemental Figure 1). One patient (C45, diagnosed with the nonprogressive hepatic subtype in childhood and then APBD in adulthood) had postmortem liver tissue examined at age 47.8 years, which demonstrated bridging fibrosis and cirrhosis (Supplemental Figure 1); this can be compared to C45’s initial liver biopsy at age 2.5 years, which exhibited bridging fibrosis and cirrhosis (9, 10).

### Hepatic disease characterization

In addition to hepatosplenomegaly and elevated aminotransferases, several patients who ultimately underwent LT or died while on the LT waiting list had a history of esophageal varices (C4, C16, C36, C44), ascites (C4, C16, C36, C63), or hepatic synthetic dysfunction (C4, C16, C26, C36, C43, C63). The additional 9 patients (C3, C7, C9, C10, C11, C12, C13, C18, C45) were followed through at least 5 years of age without the need for LT. All of these patients (hereafter referred to as having “less severe” hepatic phenotypes) had a history of elevated aminotransferases and hepatomegaly, in addition to splenomegaly in several cases (C7, C10, C11, C12); however, none of these patients had developed varices or ascites as of their last reported follow-up.

Aminotransferases were normal at the most recent laboratory evaluation in 6 of the 8 patients with less severe hepatic phenotypes (C3, C7, C9, C11, C12, C18). These included C3 and C7, whose cases were previously described in a 1996 case series that reported their outcomes through 3.8 and 5.0 years of age, respectively (9). The current study adds over 2 decades of additional follow-up data from these patients, both of whom have remained stable without clinical evidence of overt hepatic disease progression. Patient C7’s aminotransferases normalized by 10 years of age, after which he exhibited no further evidence of hepatic involvement aside from mild splenomegaly on imaging at age 16 years. At 24.8 years of age, laboratory studies noted mildly elevated aspartate aminotransferase (AST) (68 U/L), which prompted an abdominal MRI, which again showed mild splenomegaly and a normal liver aside from a benign-appearing hepatic cyst. Subsequent laboratory studies at age 25.2 years were normal. Patient C3 has likewise been followed through 29 years of age without evidence of ongoing hepatic involvement, aside from findings of a stable subcentimeter hepatic hemangioma on imaging and a transient, mild elevation of aminotransferases at 24 years of age. C3 delivered a term infant following an uncomplicated pregnancy at 29 years of age without evidence of peripartum hepatic dysfunction. In addition to C3 and C7, several others (C9, C11, C12, C18) demonstrated gradual resolution of elevated aminotransferases without other evidence of overt hepatic disease progression.

Although none of the patients with less severe hepatic phenotypes were reported to have ascites or esophageal varices, 2 patients (C10, C13) developed other features that were concerning for portal hypertension. Patient C10 presented at 1.5 years of age with hepatosplenomegaly and elevated aminotransferases; he subsequently developed persistent thrombocytopenia and synthetic dysfunction beginning at 3.5 years. Patient C13 presented with hepatomegaly and elevated aminotransferases at 2.4 years of age. Concerns for possible portal hypertension emerged after he was found to have a recanalized umbilical vein with hepatofugal flow at 2.8 years of age. Unlike C10, C13 has not demonstrated persistent evidence of splenomegaly or thrombocytopenia. Aminotransferases remained mildly elevated in C10 and C13 as of their most recent laboratory evaluations at 4.5 and 4.9 years of age, respectively.

### Extrahepatic disease characterization

#### Cardiac manifestations.

Three patients developed clinical evidence of cardiac dysfunction (C4, C6, C8) after LT, though the timing of cardiac disease progression varied among these patients (Figure 1). Patients C6 and C8 retained normal cardiac function for years after LT, whereas C4 — whose pretransplant systolic function was reportedly normal with only mild cardiomegaly — exhibited a rapid decline in cardiac function within 3 months of LT, leading to death at 1.7 years of age. By contrast, C6 did not exhibit signs of cardiac dysfunction until 11.4 years of age, when an echocardiogram showed right ventricular enlargement and impaired diastolic function. Likewise, C8 remained relatively stable until late adolescence, when he developed atrial fibrillation that was later complicated by a cerebellar stroke, followed by onset of severe systolic dysfunction (ejection fraction 10%–15%) and placement of an automatic internal cardiac defibrillator at 25 years of age.

Myocardial biopsy data were available for 3 patients (Table 1). Cardiac biopsies were performed before LT for C4 (age 1.1 years) and C5 (age 1.2 years) and more than 20 years after LT for C8 (age 24.2 years). Patients C4 and C8 exhibited clinical or imaging evidence of cardiac involvement at the time of biopsy and had histopathological evidence of cardiomyocyte hypertrophy and periodic acid–Schiff–positive (PAS-positive) inclusions. The other patient (C5) showed no clinical signs of cardiac involvement at the time of myocardial biopsy; although C5’s biopsy revealed PAS-positive inclusions in a subset of cardiomyocytes, he has since exhibited no clinical evidence of cardiac disease progression and has retained normal cardiac function through his most recent evaluation at 13.1 years of age.

#### Neurologic and muscular manifestations.

Neurologic and muscular manifestations were common in this cohort. One patient (C9) presented at birth with arthrogryposis and global hypotonia, and several others exhibited gross motor delays during the first years of life (C4, C5, C6, C26, C32, C66). Of those who underwent LT, 3 of the surviving patients reported decreased exercise tolerance compared with peers (C1) or a progressive decline in walking endurance (C5, C6) in the years following transplantation. Similar symptoms were reported in several patients with less severe hepatic phenotypes. These included C12 and his younger brother, C11, both of whom exhibited impaired muscular endurance necessitating intermittent use of a wheelchair as of their most recent clinical evaluations at 6.1 (C11) and 8.9 (C12) years of age. Likewise, C18, who presented with gait disturbance, hyperlordosis, and fatigability beginning around 2 years of age, showed persistent neuromuscular impairments (creatine kinase [CK] levels normal) when last evaluated at 8.3 years of age. Last, C45 presented with signs of APBD in his 30s.

Skeletal muscle biopsies were performed in 4 patients. Patients C5, C12, and C18 exhibited clinical evidence of neuromuscular dysfunction, such as gross motor delay, gait disturbance, and impaired endurance, at the time of biopsy. The other patient (C1) showed no clear signs of neuromuscular involvement at that time aside from a mildly elevated CK value at 1.5 years of age (368 U/L), though he later developed mildly decreased exercise tolerance in the years following LT. Notably, when reviewing the skeletal muscle pathology reports, none showed clear evidence of intramuscular polyglucosan body deposition and other histology findings were variable.

#### Other clinical findings.

In addition to the hepatic, cardiac, and neuromuscular manifestations described above, several other clinical features were shared by multiple patients. Several patients in this cohort developed laboratory and/or imaging signs of pancreatic inflammation, including C4, who had an enlarged, echogenic pancreas on ultrasound at the time of presentation; C6, who had recurrent episodes of pancreatitis between 9 and 12 years of age; C8, who was noted to have multiple instances of modest lipase elevations (up to 206 U/L) during the third decade of life; and C18, who presented with elevated lipase (1,180 U/L) and imaging evidence of pancreatic inflammation at 4.5 years of age. In addition, several patients had a history of recurrent deep vein thromboses (C4, C5, C6, C8), though these occurred in association with central lines in most cases. Other symptoms reported by multiple patients in this study included recurrent headaches (C1, C5, C12) and chronic pain in multiple extremities or joints (C3, C6, C11, C12).

Upper gastrointestinal (GI) tract complaints were also common in this cohort, with several patients (C1, C3, C5, C11, C12, C18) reporting chronic epigastric pain, dyspepsia, nausea, or other related symptoms. Patient C11 required placement of a gastrostomy tube at 2.4 years of age because of recurrent episodes of emesis. That patient’s older brother (C12) did not require gastrostomy tube placement but developed abdominal pain and hematochezia at 8 years of age. Patient C18 was found to have severe gastric ulcerations and pyloric edema on an endoscopy at 4 years of age that was obtained because of abdominal pain, distension, and emesis; her course was ultimately complicated by gastric scarring and perforation, and a comprehensive workup failed to identify a clear etiology. Although endoscopic evaluations of the remaining patients showed no gross abnormalities, biopsies of the upper and lower GI tract were available from several of these patients. Notably, C1 and C11 were found to have an increased number of histiocytes with PAS-positive, partially diastase-resistant inclusions in the lamina propria of the stomach or colorectal mucosa. Other biopsy findings that were reported included mild antral gastritis (C3), an eosinophil-rich inflammatory infiltrate in the gastric antrum (C5), eosinophilic esophagitis and fibrosis of the antral lamina propria (C10), and inactive chronic gastritis with mild inflammation of the lamina propria along with an ill-defined granuloma in the rectum (C11).

### Gbe1ys/ys mice exhibit liver involvement

**Figure 3:**
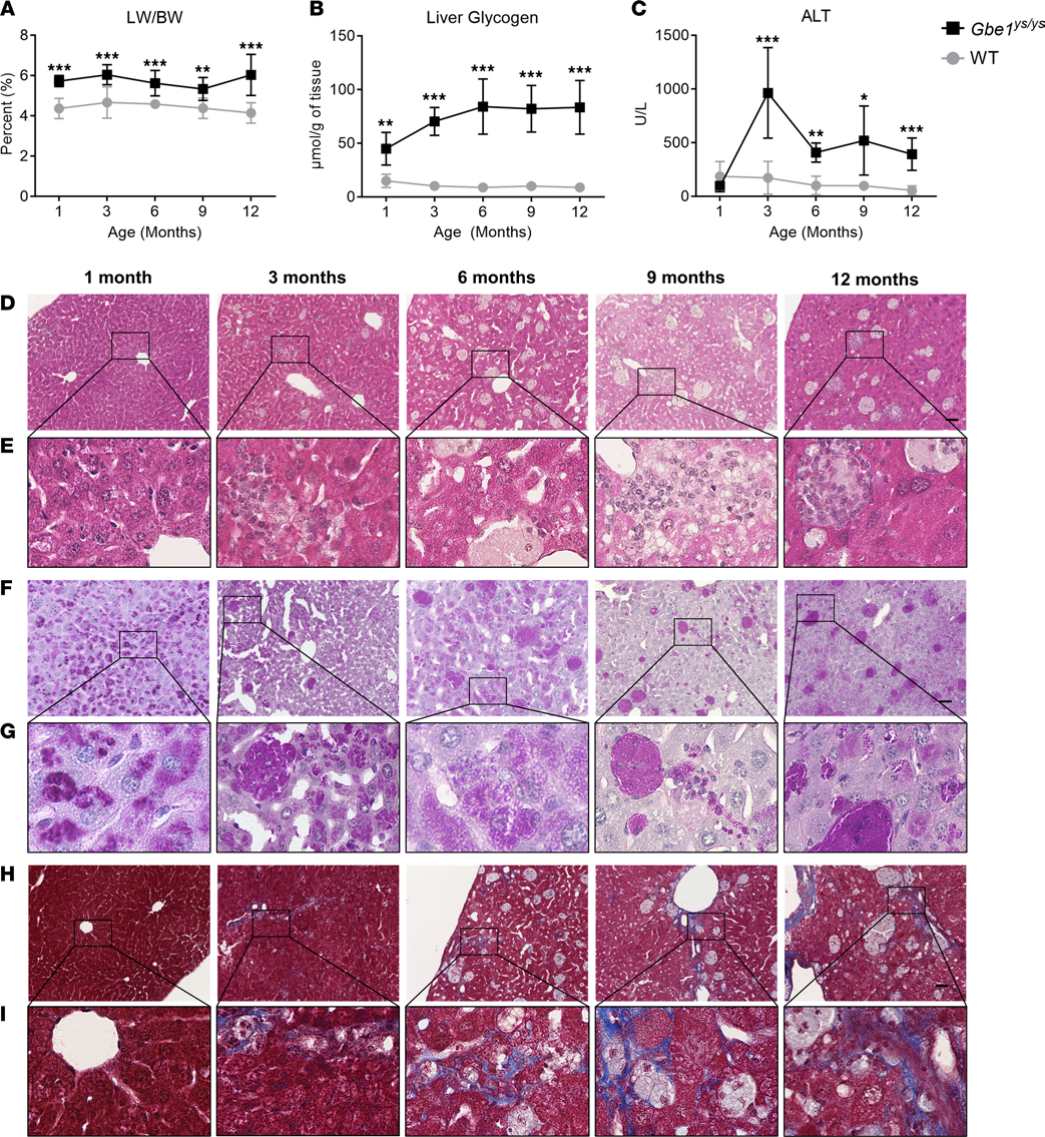
*Gbe1^ys/ys^* mice exhibit liver involvement. (**A**) Liver weight (LW) was normalized to body weight (BW) as a quantitative marker of hepatomegaly. LW/BW was significantly increased in *Gbe1^ys/ys^* mice at all time points compared with wild-type (WT) levels (*Gbe1^ys/ys^*: 1-month *n* = 5, 3-month *n* = 9, 6-month *n* = 8, 9-month *n* = 8, 12-month *n* = 7; WT: 1-month *n* = 8, 3-month *n* = 7, 6-month *n* = 7, 9-month *n* = 9, 12-month *n* = 7). (**B**) Glycogen content in the liver was significantly elevated in *Gbe1^ys/ys^* mice at all time points compared with WT levels (*Gbe1^ys/ys^*: *n* = 5 at each time point; WT: 1-month *n* = 5, 3-month *n* = 5, 6-month *n* = 5, 9-month *n* = 4, 12-month *n* = 5). (**C**) Serum alanine aminotransferase (ALT) levels were significantly elevated in *Gbe1^ys/ys^* mice compared with WT levels starting at 3 months of age (*Gbe1^ys/ys^*: 1-month *n* = 5, 3-month, *n* = 5, 6-month *n* = 5, 9-month *n* = 9, 12-month *n* = 9; WT: 1-month *n* = 5, 3-month *n* = 7, 6-month *n* = 4, 9-month *n* = 4, 12-month *n* = 5). Data presented as mean ± SD. Parametric unpaired *t* tests (2 tailed) were used to determine differences between *Gbe1^ys/ys^* and WT mice. *indicates *P* < 0.05 (considered statistically significant), **indicates *P* < 0.01, ***indicates *P* < 0.001. (**D**) Representative images of liver histology slides stained with H&E. (**E**) Zoomed view of black box region in corresponding image from **D**. (**F**) Representative images of liver histology slides stained with periodic acid–Schiff with diastase treatment. (**G**) Zoomed view of black box region in corresponding image from **F**. (**H**) Representative images of liver histology slides stained with Masson’s trichrome. (**I**) Zoomed view of black box region in corresponding image from **H**. Scale bar for panels **D**, **F**, and **H** = 50 μm and for panels **E**, **G**, and **I** = 250 µm.

*Gbe1^ys/ys^* mice were evaluated at 1, 3, 6, 9, and 12 months of age (Figure 3). Liver weight/body weight ratio was increased in *Gbe1^ys/ys^* mice (~5%–6%) compared with WT control (~4%), and liver glycogen content was significantly increased at all time points. ALT levels were significantly increased starting at 3 months, with an initial peak at 3 months of age (963 ± 421 U/L), followed by a decrease and stabilization of ALT levels above the upper limit of normal between 6 and 12 months of age (407 ± 89 to 519 ± 321 U/L). Evaluation of liver histology with PAS staining and diastase treatment revealed widespread diastase-resistant accumulations in hepatocytes as early as 1 month of age, visualization of some diastase-resistant aggregates at 3 months and 6 months of age, and then clear aggregation of diastase-resistant material at 9 and 12 months of age. The aggregates were presumably in macrophages or histiocytic collections and were associated with lobular inflammation and acidophil bodies visualized on H&E staining, the latter being a pattern that was not observed in the patient liver histology. Masson’s trichrome staining demonstrated no fibrosis at 1 month of age, foci of fine lobular fibrosis visualized around the aggregates at 3 months of age, increased frequency of foci of fine lobular fibrosis around aggregates between 6 and 12 months of age, and progression to arks of lobular fibrosis away from aggregates by 12 months of age. Quantitative measurement of liver fibrosis is provided in Supplemental Figure 2.

## Discussion

GSD IV is an autosomal recessive disorder caused by biallelic pathogenic variants in *GBE1*, which leads to deficiency of GBE and the accumulation of polyglucosan inclusions in multiple tissues. Patients with GSD IV who present with liver involvement have historically been divided into either the classic (progressive) hepatic subtype — characterized by progressive hepatic dysfunction that is fatal within the first 5 years of life without LT — or the nonprogressive hepatic subtype. Due to the challenges involved in studying this ultrarare, phenotypically heterogeneous disorder, there is a dearth of natural history data to guide clinical decision-making, especially as it pertains to the selection of appropriate candidates for LT and the long-term surveillance of both transplanted and nontransplanted patients. This study conducted a hybrid analysis of data from a phenotypically diverse cohort of patients with GSD IV and from the *Gbe1^ys/ys^* mouse model to characterize the longitudinal trajectory of hepatic disease progression in this disorder. This multinational natural history study of hepatic disease in GSD IV is the largest of its kind to date. Based on our findings, we propose a revision to the terminology that has historically been used to classify hepatic involvement in GSD IV.

### Hepatic manifestations.

This study captured clinical data from a phenotypically diverse cohort of patients with GSD IV, representing a wide range of hepatic phenotypes. The most severely affected patients presented during infancy with hepatosplenomegaly and elevated aminotransferases, accompanied by clear evidence of portal hypertension, synthetic dysfunction, or hyperbilirubinemia that typically worsened within the first 2–3 years of life. At the other end of the spectrum, several patients in this cohort presented with hepatomegaly and elevated aminotransferases in the first few years of life that later resolved within the first decade of life without the need for LT. Notably, 3 of these patients — C3, C7, and C45 — were first described in 1996, at which time they were among the first reported cases of the so-called nonprogressive hepatic subtype of GSD IV (9, 12). The current study included more than 2 decades of additional follow-up data from C3 and C7, both of whom remained stable well into the third decade of life without clinical evidence of overt hepatic disease progression. Several other patients in this cohort (C11, C12, C13, C18) appear to be following a similar clinical course, though their duration of follow-up is limited by their younger age at their most recent follow-up (range: 5.1 to 8.9 years old). C45 developed symptoms consistent with APBD in his 30s and died at age 47.8 years.

Between the phenotypic extremes represented by the established hepatic subtypes — i.e., classic (progressive) hepatic or nonprogressive hepatic — some patients may present with intermediate phenotypes. One such patient in the current study (C10) presented at 18 months of age with hepatomegaly and elevated aminotransferases. Around 3 years of age, he showed evidence of hepatic disease progression with worsening splenomegaly, thrombocytopenia, and mild coagulopathy. LT was considered but was ultimately deferred in favor of close clinical follow-up because of his overall clinical stability, without signs of decompensated cirrhosis as of his most recent follow-up at 5.2 years of age. Given that his presentation was not severe enough to justify LT within the first 5 years of life, this patient’s phenotype is not consistent with the classic (progressive) hepatic subtype, yet his overall clinical presentation with ongoing evidence of portal hypertension and synthetic dysfunction suggests a greater degree of hepatic disease than would be expected with the so-called nonprogressive hepatic subtype. A handful of other patients with GSD IV have been reported in the literature who appeared to exhibit similar phenotypes, characterized by onset of portal hypertension and/or synthetic dysfunction that persisted over time but was not severe enough to warrant LT during early childhood (13–15). Notably, several of these patients later developed serious hepatic complications after a period of prolonged clinical stability. These included 1 patient who required LT in the setting of hepatic deterioration at age 12 years (13), another who decompensated and died at 13 years of age with evidence of hepatocellular carcinoma (HCC) on autopsy (14), and another who underwent LT after she was found to have HCC on a surveillance ultrasound at age 21 years (15).

The existence of patients with intermediate hepatic phenotypes suggests that there is continuous variation in hepatic disease severity, challenging the division of this disorder into discrete hepatic subtypes. The breadth of this phenotypic spectrum complicates the selection of appropriate candidates for LT, which remains the only disease-modifying therapy for patients with severe hepatic phenotypes (7). Importantly, the tests that are used to support the diagnosis of GSD IV appear to be of limited utility for predicting hepatic disease severity within the GSD IV phenotypic spectrum. Several patients in this study who exhibited long-term clinical stability without the need for LT (C3, C7, C12, C13, C18) were found to have advanced fibrosis or cirrhosis on liver biopsies obtained during infancy, suggesting that severe disease at the tissue level does not, in isolation, portend a poor clinical outcome. Yet, lifelong monitoring is warranted because of potential risk for further liver disease and hepatic fibrosis progression later in life (7), as well as rare reports of hepatocellular adenoma and HCC (14–17). The prognostic value of residual GBE is likely to be similarly limited, particularly when assessed in the liver (7). Predicting hepatic disease severity based on *GBE1* genotype is challenging because of the rarity of this disorder and the high prevalence of private mutations, which complicates the effort to infer robust genotype-phenotype relationships (18). At least 1 pair of affected siblings with GSD IV have been reported to be discordant for LT status, suggesting that phenotype may differ to a clinically meaningful extent even in patients with the same biallelic *GBE1* genotype (18). In the absence of reliable prognostic markers to predict disease severity at the time of diagnosis, “prophylactic” LT before the onset of decompensated cirrhosis is unlikely to be an appropriate approach for patients with GSD IV. Among patients with GSD IV who do not yet exhibit clear evidence of progressive hepatic dysfunction, proceeding with preemptive LT carries the risk of subjecting those who would otherwise have developed less severe hepatic phenotypes to unnecessary transplant-related morbidity. Thus, a period of careful clinical surveillance may be a better alternative to immediate LT for select patients with hepatic GSD IV; this recommendation is consistent with published clinical practice guidelines for the management of GSD IV (7).

For patients with less severe hepatic phenotypes that do not require immediate LT, the mechanisms by which overt clinical manifestations of hepatic involvement may resolve over time, despite history of biopsy-proven fibrosis or even cirrhosis, are not well understood. Specifically, it remains unknown whether the clinical improvement is coupled to regression of hepatic fibrosis at the tissue level or if patients exhibit long-term persistence of stable or progressive hepatic fibrosis despite their apparent clinical stability. In this study, we report on the autopsy liver pathology from an individual (C45) diagnosed with nonprogressive hepatic GSD IV at age 2.5 years who later died at age 47.8 years. On initial liver biopsy at age 2.5 years, bridging fibrosis and cirrhosis was noted (9, 10). Liver pathology from the autopsy decades later demonstrated some PAS-positive diastase-resistant inclusions as well as bridging fibrosis and cirrhosis, confirming that fibrosis persisted and did not regress. The potential for such underlying “silent” fibrosis should be considered when counseling affected families and coordinating long-term follow-up. Moreover, several cases with less severe hepatic phenotypes developed splenomegaly without thrombocytopenia (C7, C11, C12); though this could be suggestive of possible portal hypertension, we cannot rule out if the spleen enlargement was a result of a separate disease sequelae, such as macrophages accumulating polyglucosan in the spleen.

Future work should investigate the pattern of hepatic fibrosis in individuals who progress rapidly versus those who survive without LT to identify if there are differences in the pathophysiology of their fibrosis progression or the contribution of other genomic factors. Although the validity of hepatic elastography — a noninvasive imaging modality that measures tissue stiffness to estimate the severity of hepatic fibrosis — has been studied in other childhood-onset liver disorders (19–21), limited data are available to guide the application of this technique to patients with GSD IV. This study included hepatic elastography data from 4 patients (C10, C12, C16, C18; Figure 2) whose phenotypes collectively spanned the full spectrum of hepatic disease severity. Patient C16, who exhibited a severe phenotype leading to death at age 2 years, showed severely increased liver stiffness (66 kPa) on a transient elastography study at 1.7 years of age. For the 3 other patients, all of whom exhibited less severe hepatic phenotypes, their most recent elastography studies showed either normal (C12, C18) or only borderline increased liver stiffness (C10; median 1.65 kPa). These findings, if accurate, could suggest longitudinal regression of hepatic fibrosis in C10, C12, and C18, all of whom had advanced fibrosis or cirrhosis on their baseline liver biopsies. Nonetheless, these findings should be interpreted with caution since there were at least 2 instances in which the elastography findings were challenging to reconcile with other data; these included patient C10’s elastography study at 4.5 years of age, which showed apparently normal liver stiffness even though concurrent laboratory studies revealed thrombocytopenia and mildly prolonged INR, and patient C18’s elastography study at 5.3 years of age, which showed normal stiffness less than 1 year after her liver biopsy had revealed bridging fibrosis to cirrhosis. Given these findings, the possibility that elastography may have underestimated the extent of hepatic fibrosis in these patients cannot be excluded. Studies comparing liver biopsy findings to concurrent elastography measurements are needed to validate the accuracy of this imaging technique in patients with GSD IV, particularly for those with less severe hepatic phenotypes. Until comprehensive elastography data in GSD IV are gathered, caution should be taken when interpreting elastography results alone. These results should instead be viewed in the context of the full clinical picture.

Given the challenges involved in studying long-term changes in hepatic fibrosis in human patients, animal models may provide important additional insights into this process. This study analyzed longitudinal histopathological data from the *Gbe1^ys/ys^* mouse model, a homozygous knockin for the pathogenic *Gbe1* variant p.Y329S, which has traditionally been used in APBD research (11, 22–26). Previous reports of 4-month-old *Gbe1^ys/ys^* mice demonstrated polyglucosan accumulation and perisinusoidal/pericellular fibrosis in the liver (11). With deeper phenotyping, we uncovered that clinically, this model exhibits features of both the less severe hepatic phenotype of GSD IV (liver fibrosis, hepatomegaly, and elevated ALT without progressive liver failure) and APBD. This is consistent with the observation that human patients with the p.Y329S variant in compound heterozygosity with another *GBE1* variant typically present with less severe hepatic phenotypes in childhood (including C3, C11, C12, and C45) and/or neurological features consistent with APBD (10, 12, 18, 27). In this study, the analysis of histopathological data from *Gbe1^ys/ys^* mice evaluated over 5 time points showed that lobular fibrosis progressed during the first 6 months of age and stabilized thereafter, persisting through 12 months of age. Similarly, levels of liver glycogen (presumably polyglucosan) (28) increased during the first 6 months and then stabilized through 12 months of age. These findings raise the possibility that hepatic fibrosis may persist over time in GSD IV patients with less severe hepatic phenotypes. However, whereas all patients exhibited extensive bridging fibrosis or cirrhosis on liver biopsy early in life, the *Gbe1^ys/ys^* mice did not develop fibrosis to that degree. Moreover, whereas ALT remained elevated through 12 months of age (equivalent of adulthood) in *Gbe1^ys/ys^* mice, ALT normalized within the first decade of life for several patients in the current study (including C3, C7, C9, C11, C12, and C18). Thus, it is unclear to what extent this mouse model recapitulates the phenotype of patients on the GSD IV hepatic disease spectrum. Given these uncertainties about the longitudinal trajectory of hepatic fibrosis in GSD IV, long-term hepatic surveillance is warranted for all patients, including those who do not require LT during early childhood.

### Extrahepatic manifestations.

Independent of the severity of hepatic disease, the potential for cardiac and/or neuromuscular manifestations to progress over time — up to decades after the initial presentation, in some cases — further complicates the management of patients with GSD IV. Although early studies suggested that LT might ameliorate or prevent extrahepatic disease progression among patients with GSD IV (29), this view has since been challenged (16, 30, 31). The current study included 3 patients who developed cardiac dysfunction following LT, which became apparent during the peritransplant period in 1 case (C4) and did not emerge until years after transplantation for 2 others (C6, C8). Although none of the patients with less severe hepatic phenotypes in this cohort have developed cardiac dysfunction to date, there have been at least 2 published cases of patients with GSD IV who reportedly presented with nonsevere hepatic phenotypes during childhood (permitting survival to adulthood without LT) and later developed cardiomyopathy during the second or third decade of life (32, 33). These findings support the value of long-term cardiac surveillance of all patients with GSD IV, regardless of hepatic disease severity. Neuromuscular involvement — which manifested in childhood/adolescence with findings such as decreased exercise tolerance, gait disturbance, hyperlordosis, gross motor delays, and other abnormalities — was also common in this cohort, affecting patients with severe (C1, C4, C5, C6, C26, C32, C66) as well as less severe (C9, C11, C12, C18) hepatic phenotypes.

Predicting which patients with GSD IV are likely to develop extrahepatic involvement is challenging, in part because the prognostic value of cardiac and skeletal muscle biopsies appears to be limited. In the current study, patient C5 was found to have polyglucosan inclusions on a cardiac biopsy at 15 months of age, yet he retained normal cardiac function through his most recent evaluation at age 13 years. Conversely, abnormal glycogen deposition in skeletal myocytes was not demonstrated on any of the skeletal muscle biopsies from this cohort, even though all 4 patients showed either clinical (C5, C12, C18) or laboratory (C1; elevated CK) evidence of neuromuscular involvement around the time of biopsy. It is unclear whether this reflects a sampling phenomenon or if factors other than intramuscular polyglucosan accumulation may contribute to neuromuscular dysfunction in these patients. Regardless, these findings suggest that the identification of polyglucosan bodies on histopathology does not guarantee clinical dysfunction of a given organ and that their absence does not preclude it. Despite these limitations, tissue biopsies may still be valuable for excluding other comorbid pathologies in these patients (7).

Separate from the childhood-onset neuromuscular manifestations that were reported in several patients in this cohort, there is emerging evidence that patients with hepatic GSD IV may also be at risk for developing a related, but distinct, constellation of adult-onset neurological disturbances associated with APBD. These include neurogenic bladder, peripheral neuropathy, spasticity, muscle weakness, and other signs of both upper and lower motor neuron dysfunction. Although GSD IV and APBD were previously believed to represent distinct allelic disorders with onset in childhood and adulthood, respectively (7), several lines of evidence support the possibility that features of both conditions may occur within the same patient. Notably, patient C45 was diagnosed with nonprogressive hepatic GSD IV in childhood as described previously (9), and he was later reported to have developed APBD during the fourth decade of life (10). The potential overlap between hepatic GSD IV and APBD is also supported by findings from the *Gbe1^ys/ys^* model, which, as noted previously, exhibits features of both APBD and the less severe hepatic phenotype of GSD IV (34). Additionally, there is genotypic overlap between GSD IV and APBD, as evidenced by the observation that the *GBE1* genotype of patient C3 (c.986A>C and c.671T>C) has also been reported in patients with APBD (27, 35). Similarly, patients C1 and C10 were found to share the same *GBE1* genotype (c.691+2T>C and c.1544G>A) as a patient who was diagnosed with APBD after presenting with acute-onset spastic quadriparesis at 65 years of age (36). Given these findings, more research is needed to characterize the relationship between hepatic GSD IV and APBD.

Overall, the findings of the current study are consistent with published recommendations for the care of patients with GBE deficiency, which underscore the need for all patients with this disorder to be monitored longitudinally for evidence of hepatic, cardiac, muscular, and neurologic disease progression, including signs of APBD (7). Moreover, in addition to the established sequelae of GBE deficiency, there may be other aspects of the phenotypic spectrum that have not yet been well characterized. Notably, several patients in this cohort developed signs and symptoms of unclear etiology, such as chronic nausea, epigastric discomfort, or gastritis (C1, C3, C5, C11, C18); recurrent thromboses (C4, C5, C6, C8); laboratory or imaging evidence of pancreatitis (C4, C6, C8, C18); joint pain (C3, C6, C11, C12); and recurrent headaches (C1, C5, C12). Due to the rarity of this disorder, it is challenging to determine whether these findings were incidental or if they reflect underrecognized manifestations of GBE deficiency. Another important area for future research concerns the pathogenesis of hypoglycemia in GSD IV, which was observed in several patients with otherwise intact liver function (C9, C11, C12). Additional natural history studies are needed to address these and other unanswered questions. Last, the effect of dietary intervention on the progression of GSD IV–related liver disease or polyglucosan accumulation warrants further investigation. Previous papers have reported the effects of various dietary regimens on the clinical outcomes of patients with hepatic GSD IV (18, 37–39), yet universal dietary recommendations for patients with GSD IV have not been established (7).

### A proposed revision of descriptive terminology for hepatic GSD IV.

Collectively, the findings of the current study highlight the limitations of the established subtype-based approach to characterizing hepatic disease in GSD IV. The validity of discrete classic (progressive) hepatic and nonprogressive hepatic subtypes is undermined by the existence of patients with intermediate phenotypes, who may exhibit stable or slowly progressive portal hypertension that persists over time but is not severe enough to warrant LT within the first 5 years of life. Even for patients at the least severe end of the hepatic disease spectrum — including those for whom all clinical and laboratory signs of hepatic disease resolve within the first decade of life — the use of the descriptor “nonprogressive” is potentially misleading since these patients may continue to be at risk for developing hepatic and extrahepatic disease manifestations, such as neurologic or muscular involvement, cardiomyopathy, and APBD. Additionally, the possibility that hepatic fibrosis may persist in these patients is supported by findings from both the *Gbe1^ys/ys^* model and C45’s postmortem liver examination. Given these considerations, it may be more accurate to characterize these patients as exhibiting attenuated hepatic disease, rather than a nonprogressive phenotype.

**Figure 4:**
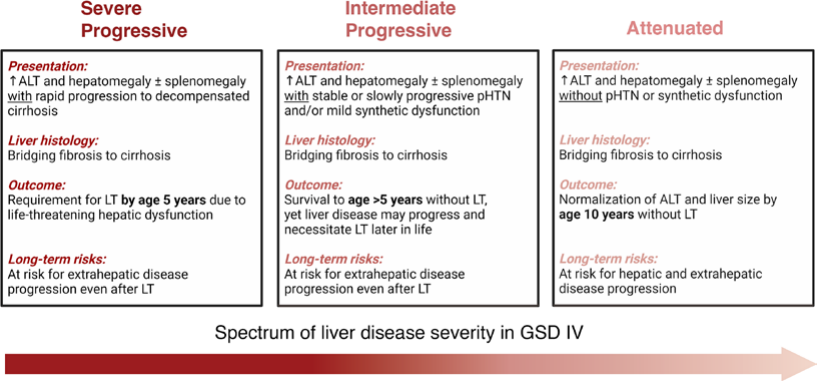
Proposed alternative to the established subtype-based terminology for characterizing liver disease in GSD IV. Typical symptoms at presentation, liver histology findings, outcomes, and long-term risks are detailed for each proposed category. ALT, alanine aminotransferase; LT, liver transplantation; pHTN, portal hypertension.

Based on these findings, we propose an alternative to the established subtype-based terminology for characterizing liver disease in GSD IV (Figure 4). Our proposed system recognizes 3 tiers of severity that better capture the full spectrum of hepatic disease in GSD IV. These include (i) “severe progressive” hepatic disease, characterized by rapidly progressive hepatic dysfunction, portal hypertension, and decompensated cirrhosis that necessitates LT within the first 5 years of life; (ii) “intermediate progressive” hepatic disease, characterized by the presence of portal hypertension that is persistent or slowly progressive but does not necessitate LT within the first 5 years of life; and (iii) “attenuated” hepatic disease, characterized by elevated ALT levels and hepatomegaly that resolve over time — typically by the first 10 years of life — without portal hypertension. The term “attenuated” is preferred over “nonprogressive” in this context because it better accounts for the possibility that stable or ongoing subclinical hepatic injury or extrahepatic disease progression may occur even in otherwise-asymptomatic patients.

Ideally, reliable genotype-phenotype correlations would be identified to aid in prognosis and prediction of clinical outcomes for patients with hepatic GSD IV. Previous groups have attempted to delineate genotype-phenotype correlations (40–42), but no clear relationships for hepatic GSD IV have been established, largely due to limited natural history data and the increasing number of causative *GBE1* variants. The c.986A>C variant in homozygosity has a well-documented association with APBD, but when in compound heterozygosity, the prognosis is unclear. We describe 3 patients (C3, C11, C12) who are heterozygous for the c.986A>C variant and who all have followed a less severe/attenuated hepatic disease course, but the young age of C11 (6.1 years) and C12 (8.9 years) restricts the ability to draw any conclusions about long-term outcomes. Additionally, the c.691+2T>C and c.1544G>A variants were observed in compound heterozygosity in 4 patients (C1, C9, C10, C13) and 6 patients (C1, C4, C5, C10, C44, C63), respectively; both variants were observed in patients on all ends of the hepatic disease spectrum: severe progressive, intermediate progressive, and attenuated hepatic disease. Despite not being able to elucidate clear genotype-phenotype relationships, the genotype data from our cohort suggest that a less severe hepatic disease course (i.e., intermediate progressive or attenuated) does not occur in individuals with 2 null *GBE1* variants. Continuing to publish on the natural history of the disease is imperative to improve our ability to predict clinical outcomes. Regardless of expected prognosis, all patients should receive close and lifelong monitoring because of potential for ongoing subclinical liver disease and risk for further hepatic and extrahepatic disease progression (7).

This study yielded several key insights into the natural history of hepatic disease in GSD IV based on joint analysis of data from a phenotypically diverse cohort of patients and a knockin mouse model. Our findings challenge the established subtype-based view of this disorder and suggest that there is continuous variation in hepatic disease severity among patients with GSD IV. The breadth of this phenotypic spectrum, combined with the limited prognostic value of liver biopsy, complicates clinical decision-making and the selection of appropriate candidates for LT. Moreover, although a subset of patients in this study were shown to exhibit near-complete resolution of all clinical evidence of hepatic involvement without the need for LT, findings from the *Gbe1^ys/ys^* model and the autopsy of an individual previously diagnosed with nonprogressive hepatic GSD IV support the possibility that subclinical hepatic fibrosis might persist in these patients, underscoring the need for careful clinical follow-up. The potential for extrahepatic disease manifestations to progress even decades after symptom onset further complicates the care of these patients. Given these challenges, key priorities for future research include the identification of prognostic biomarkers, characterization of the mechanisms that contribute to divergent hepatic disease trajectories in this disorder, and clarification of the relationship between hepatic GSD IV and APBD. A detailed understanding of the longitudinal progression of this disorder is needed to inform clinical management, identify therapeutic endpoints, and evaluate the efficacy of treatments as they become available.

## Methods

This study characterized the natural history of hepatic disease in GSD IV using a dual approach that consisted of (i) a retrospective, longitudinal analysis of clinical data — including laboratory, histopathology, and imaging studies — from a multinational cohort of patients with GSD IV and (ii) a prospective study of longitudinal changes in serum ALT levels, liver glycogen content, and liver histopathology in the *Gbe1^ys/ys^* mouse model.

### Sex as a biological variable

Our study examined male and female patients and mice.

### Patient data

#### Inclusion criteria and enrollment.

To be included in the current analysis, patients were required to have 1) received a diagnosis of GSD IV based on the identification of biallelic *GBE1* variants, deficient GBE activity, and/or characteristic histopathological findings on liver biopsy and 2) exhibited clinical evidence of hepatic involvement, defined as any current or prior history of elevated aminotransferases, hepatomegaly, splenomegaly, varices, ascites, and/or synthetic dysfunction. Each patient was assigned a unique identification number preceded by the letter *C* (for “case”). Identification numbers corresponded to enrollment number in the study.

#### Data extraction.

For each eligible patient, clinical data were either extracted from their original medical records (released with the written permission of the patient or authorized representative after informed consent was obtained) or from deidentified summaries provided by the patient’s treating clinician. Standardized data entry forms were used to extract relevant information about each patient’s clinical course and any other pertinent clinical history. Study data were collected and managed using Research Electronic Data Capture electronic data capture tools hosted at Duke University (43). For each patient, a detailed narrative summary of their clinical course was generated and is reported in Supplemental Data 1.

#### Analysis of laboratory data.

Laboratory parameters ALT and INR were selected as noninvasive indices of hepatocellular injury and synthetic function, respectively (44). If the parameter of interest was measured more than once during a given interval, the available values were averaged. ALT values were expressed as multiples of the upper limit of normal given in the original laboratory report. If the original reference range was unavailable, published sex- and age-adjusted norms were used (45).

#### Evaluation of liver fibrosis.

Liver fibrosis was characterized using a combination of invasive assessments (liver biopsy conducted as part of clinical care) and noninvasive measures (elastography and conventional imaging studies). Elastography is a noninvasive imaging modality — encompassing techniques such as transient elastography, acoustic radiation force impulse imaging, shear wave elastography, and magnetic resonance elastography — that evaluates the extent of hepatic fibrosis based on quantitative measurements of liver stiffness. Prior studies have compared liver biopsy findings to contemporaneous elastography studies in order to establish optimal cut points for predicting fibrosis stage (METAVIR F0–F4) from elastography measurements (46–48). In the current study, all available elastography results — originally reported in units of meters per second (shear wave velocity) or kilopascals (liver elastic modulus) — were coded into 1 of 3 categories: normal liver stiffness (METAVIR equivalent F0), borderline or mildly increased stiffness (METAVIR equivalent F1), or moderately to severely increased stiffness (METAVIR equivalent F2–F4). These categories were designed to encompass the definition of “clinically significant fibrosis,” which includes METAVIR stage ≥ F2 (47). Each available elastography result was classified into one of these groups based on the reference ranges given in the original imaging report (if available) or using published thresholds for METAVIR equivalents (46–50). The use of this trilevel classification system allowed for results from different elastography modalities to be compared over time.

#### Scoring of conventional abdominal imaging studies.

Finally, the radiology reports of conventional abdominal imaging studies — such as ultrasound (USG), CT, and MRI — were systematically scored based on the presence of (i) hepatic abnormalities (liver surface or parenchyma) consistent with fibrosis or cirrhosis and (ii) signs of clinically evident portal hypertension (CEPH). For the purposes of the current study, the appearance of the liver was scored as abnormal if the radiology report noted any of the following: surface nodularity, increased echogenicity on USG, heterogeneous echotexture on USG or enhancement on MRI, or blunting of the liver edge (51, 52). CEPH was defined as presence of varices, ascites, or splenomegaly with thrombocytopenia (platelets < 150,000, measured within 3 months of the index imaging study) (53).

### Gbe1ys/ys mouse model

#### Mice and housing conditions.

The *Gbe1^ys/ys^* mouse colony was provided by W. J. Craigen and H. O. Akman at Baylor College of Medicine (11). *Gbe1^ys/ys^* and WT C57BL/6 mice were bred and genotyped as previously described (34). All animals were housed at Duke University and provided standard chow and water ad libitum. Mice were sacrificed at 1, 3, 6, 9, or 12 months of age. Mice were fasted for 24 hours, and blood was collected via the inferior vena cava prior to sacrifice. Body and liver weight were documented immediately after sacrifice.

#### Glycogen content assay.

Liver was homogenized in distilled water (1 mg of tissue/20 μL of water) using a homogenizer, followed by sonication for 1 minute (pulse 20 seconds, pause 10 seconds with amplitude 20%). To dissolve the insoluble glycogen, the samples were boiled at 100°C for 5 minutes (54), cooled down at room temperature, and centrifuged at 16,000*g* at 4°C for 20 minutes. The supernatant was used for quantification of glycogen content. The 1:5 diluted lysates were incubated with 0.175 U/mL (final concentration in the reaction) of amyloglucosidase (MilliporeSigma) for 90 minutes in a 37°C water bath. The reaction mixtures were then boiled again for 3 minutes to stop the reaction and centrifuged at 16,000*g* at 4°C for 3 minutes. Then, 30 μL of the mixtures were incubated with 1 mL of Pointe Scientific Glucose (Hexokinase) Liquid Reagents (Thermo Fisher Scientific) for at least 10 minutes at room temperature. The absorbance was read at 340 nm using a UV-VIS spectrophotometer (Shimadzu UV-1700 PharmaSpec).

#### Laboratory tests.

Blood was collected via the submandibular vein in a red top collection tube and centrifuged at 2,000*g* at 4°C for 10 minutes to isolate serum. Serum was evaluated for ALT (U/L) activity according to the manufacturer’s manual for ALT (Pointe Scientific, Liquid ALT Reagent Set A7526). Briefly, the 1:5 diluted serum with 0.9% NaCl was added to the reaction reagent and incubated at 37°C water bath for 1 minute. Then, the absorbance was read using a UV-VIS spectrophotometer. Repeat readings were recorded every minute for 4 minutes and were averaged to calculate mean absorbance difference/min.

#### Histology.

Sections of the liver were isolated and fixed in 10% neutral-buffered formalin (NBF), then embedded in paraffin, and sections were stained with PAS with diastase treatment, H&E, and Masson’s trichrome as previously described (55). Briefly, the tissues were fixed in 10% NBF for 48 hours, and then samples were postfixed with 1% periodic acid (PA) in 10% NBF for 48 hours at 4°C. The fixed samples were washed with PBS and dehydrated with ascending grades of alcohol, cleared with xylene, and infiltrated with paraffin. For staining with PAS with diastase treatment, slides were deparaffinized and hydrated and then incubated in fresh 0.5% amylase in a 37°C water bath for 20 minutes and rinsed with tap water. The slides were oxidized with freshly made 0.5% PA for 5 minutes and stained with Schiff reagent for 15 minutes. Then slides were counterstained with hematoxylin and incubated with bluing solution for 1 minute. For H&E staining, slides were stained with hematoxylin for 8 minutes, washed with running tap water, and then counterstained in eosin Y solution for 3 minutes. For Masson’s trichrome staining, slides were stained with Masson’s trichrome stain kit HT15 (MilliporeSigma). Slides were incubated in Bouin’s solution at room temperature overnight, washed with distilled water, and then stained with Weigert’s Iron hematoxylin solution for 5 minutes. Slides were rinsed with distilled water and placed in phosphotungstic/phosphomolybdic acid solution for 5 minutes, washed with distilled water, incubated in Aniline blue solution for 5 minutes, and rinsed again. Slides were placed in 1% acetic acid for 2 minutes, dehydrated, and mounted. The images were taken using a BZ-X710 microscope (Keyence America).

### Histopathology review

Of the 23 patient cases, 19 had liver biopsy information available. Patient C45 had additional liver tissue collected during autopsy. All mouse liver histology slides and available liver histology slides or images from 7 patients were reviewed by a board-certified pathologist with expertise in hepatic GSD with genotype and age blinded (Supplemental Figure 1). For the remaining 12 patients without available slides or images, data were extracted from the original pathology report/medical record.

### Statistics

Quantitative data were evaluated using Prism version 9 software (GraphPad) and analyzed using parametric unpaired 2-tailed *t* tests to determine differences between results in *Gbe1^ys/ys^* and WT mice. Data were reported as mean ± SD. *P* < 0.05 was considered statistically significant.

### Study approval

The Duke University Health System Institutional Review Board provided approval for all research involving human participants (Pro00060753). Written informed consent was obtained by participants who spoke English and were able to be contacted. For participants where written informed consent was not otherwise obtainable, deidentified participant data were submitted by their care providers as part of a multinational collaboration under an approved waiver of informed consent. The studies involving animals were approved by Duke University Institutional Animal Care and Use Committee (A170-21-08). The studies involving humans and animals were conducted in accordance with local legislation and institutional requirements.

### Data availability

Associated patient and mouse data are included in the Supporting Data Values file.

## Author contributions

PSK supervised the study; RLK and BTK wrote the original draft; and RLK, BTK, SJC, WRJ, LSF, VS, SA, GP, KL, and CSA performed writing, review, and editing. RLK and BTK share co–first authorship. RLK contributed to the design and implementation of the research, data analysis, interpretation, and review and editing of the manuscript. BTK contributed to the implementation of the research, data analysis, and interpretation.

## Supplementary Material

Supplemental data

ICMJE disclosure forms

Supplemental table 1

Supporting data values

## Figures and Tables

**Table 1:**
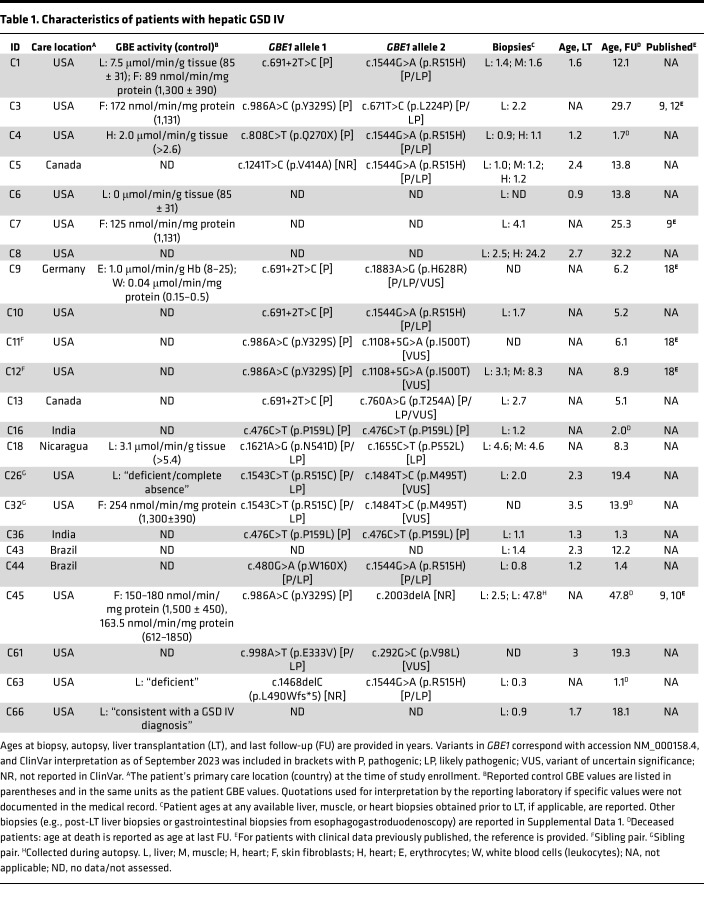
Characteristics of patients with hepatic GSD IV

## Version 1 06/24/2024

Electronic publication
