# Supplementary material for: Natural history study of hepatic glycogen storage disease type IV and comparison to Gbe1ys/ys model
Source: JCI Insight. 2024 May 14;9(12):e177722. doi: 10.1172/jci.insight.177722 (PMC11383185; doi:10.1172/jci.insight.177722)
Supplement: Supplemental data [file jciinsight-9-177722-s248.pdf]

C1

Patient C1 is an American White male of German, English, and American Indian descent who was born at 39 weeks of gestation following a normal pregnancy. The neonatal period was uncomplicated, and he had no major illnesses during the first year of life. He achieved his gross motor milestones on time, walking around 10 months of age. His developmental history was notable only for an oral aversion with refusal of all solid foods by 12 months of age, despite which his growth remained normal. At 16 months of age, he presented to the emergency department with an acute febrile illness and abdominal distension. On exam, he was noted to have hepatosplenomegaly, and laboratory studies showed elevated transaminases (AST 665 U/L, ALT 780 U/L) with normal bilirubin, coagulation studies, and albumin. An abdominal ultrasound revealed a hyperechoic liver parenchyma and splenomegaly.

These findings prompted a liver biopsy at 16 months of age, which demonstrated extensive bridging fibrosis and fibrillary, amylopectin-like material. The diagnosis of GSD IV was confirmed via identification of biallelic *GBE1* mutations and deficient GBE activity in skin fibroblasts and the liver (Table 1). An ECG and echocardiogram at 1.4 years of age were unremarkable. His serum creatine kinase (CK) was mildly elevated at 1.5 years of age (368 U/L) but he otherwise showed no evidence of neuromuscular involvement, with normal strength and tone on exam. A muscle biopsy from the right quadriceps muscle at 1.6 years of age was notable only for a slight alteration in muscle fiber glycogen content, variation in muscle fiber diameter, and increased mitochondrial activity. Electron microscopy showed a small amount of amorphous, granular, fibrillary material in the muscle stroma. This was not present within macrophages or muscle fibers, which contained morphologically normal glycogen. These histopathological abnormalities were judged to be minimal and of uncertain clinical significance, consistent with his lack of neuromuscular symptoms at the time of biopsy.

The patient underwent liver transplantation (LT) at 1.6 years of age. His immediate post-transplant course was complicated by fever, leukocytosis, and progressive abdominal distension requiring exploratory laparotomy within two weeks of the initial surgery. He developed abdominal wall cellulitis at 1.8 years of age. Over the following three years, he was hospitalized for multiple episodes of biopsy-confirmed acute cellular rejection with fever and elevated transaminases, most recently at 4.7 years of age. Thereafter, he had no further episodes of acute rejection, although he required multiple hospitalizations for febrile viral illnesses, including Epstein-Barr virus and adenovirus viremia. His most recent liver biopsy at 6.2 years of age showed only rare inflammatory cells without evidence of rejection. At 7.3 years of age, he underwent a colonoscopy and esophagogastroduodenoscopy (EGD) for abdominal pain, which was notable for findings of PAS-positive, partially diastase-resistant inclusions in macrophages of the colorectal lamina propria. Similar inclusions had also been noted in samples from a previous colonoscopy at 1.7 years of age.

As of his last follow-up at 12.1 years of age, patient C1 was stable with normal transaminases and hepatic function. His last echocardiogram at 11.0 years of age showed normal biventricular size and cardiac function. He complained of increased muscular fatigability and difficulty keeping up with peers, although his neurological exam and serum CK were normal when last assessed at 11.7 years of age. He has no other chronic issues aside from well-controlled hypertension due to chronic use of calcineurin inhibitors.

C3

Patient C3 is an American White female of Ashkenazi Jewish descent who was previously described by Bao et al. (Patient 2) and McConkie-Rosell et al. (Patient C) in a 1996 case series of patients with the so-called "non-progressive hepatic" phenotype of GSD IV. The current study reports nearly 25 years of additional follow-up data from this patient.

Patient C3 presented with elevated transaminases, hepatomegaly, and failure to thrive at 12 months of age. By 1.5 years for age, her length and weight were approximately 3 standard deviations below the mean for her age. Transaminases peaked at 1.5 years of age (AST 643 U/L, ALT 462 U/L) and began to decline thereafter, although a subsequent spike prompted the decision to pursue a liver biopsy at 2.2 years of age. This showed advanced fibrosis with portal to portal and portal to central bridging and PAS-positive deposits that were partially resistant to diastase digestion. The diagnosis of GSD IV was confirmed via measurement of deficient GBE activity in skin fibroblasts at 2.5 years of age and identification of biallelic mutations in the *GBE1* gene (Table 1). She did not exhibit signs of portal hypertension, synthetic dysfunction, or jaundice. Her liver size and transaminases gradually decreased over time and were normal at the time of her last published follow-up at 3.8 years of age. Her motor development was appropriate, and she exhibited no evidence of cardiac involvement.

This patient has since been followed through 29.9 years of age and has remained stable without clinical evidence of hepatic disease progression. She reached an adult height of 62 inches (157 cm) with a normal BMI. Transaminases have remained within normal limits, aside from a mild, transient increase at 26.0 years of age (AST 88 U/L, ALT 81 U/L), which normalized within one month. The appearance of the liver has remained normal on longitudinal imaging studies (completed at 18, 23, 25, 27, and 28 years of age) aside from a stable sub-centimeter hepatic hemangioma. She has not had splenomegaly or other signs of portal hypertension.

Although the patient had sufficient exercise tolerance to participate in dance at an elite level during adolescence and early adulthood, her activities were subsequently affected by persistent musculoskeletal pain of unclear etiology. She developed severe shoulder pain for which she underwent bilateral arthroscopic surgery at ages 25 and 26. These interventions resulted in only partial symptom improvement, with persistence of pain and intermittent right upper extremity numbness. An MRI of the right shoulder and cervical spine was non-diagnostic, and an EMG/NCS at 26.8 years of age was notable only for enlarged motor units in the first dorsal interosseous muscles, without other evidence of plexopathy or neuropathy. In addition to pain, she also reported weakness and easy fatigability of the upper extremities which interfered with her work and activities of daily living. She also developed diffuse joint pain (involving the hands, knees, hips, and back) that prompted a rheumatological evaluation, which was notable only for a borderline elevated CRP. She reported some improvement in symptoms with use of nortriptyline.

In addition to her musculoskeletal concerns, the patient has been followed for chronic gastrointestinal and genitourinary symptoms. Beginning in adolescence, she reported severe epigastric pain, early satiety, regurgitation, and retrosternal burning, for which she underwent an EGD at 18 years of age. This showed a normal-appearing gastric and duodenal mucosa aside from mild antral gastritis. Similar findings were noted on a repeat EGD at 20 years of age. CT imaging of the abdomen and pelvis was non-diagnostic aside from an incidental finding of an enlarged mesenteric lymph node, which remained stable on repeat imaging. Her symptoms were largely refractory to multiple trials of PPI and H2-blocker medications, although she reported some improvement with regular use of dicyclomine. In addition to her gastrointestinal concerns, the patient required urological evaluation at 24 years of age for persistent pelvic pain and urinary symptoms, including intermittent dysuria, urgency, and urinary frequency without incontinence. Pelvic ultrasound was unremarkable and cystoscopy showed a normal bladder mucosa. On uroflowmetry, urine flow rate was noted to be somewhat low, although this was felt to reflect pain rather than a true voiding limitation. There

was no evidence of urinary retention. The patient's symptoms were attributed to interstitial cystitis and/or pelvic floor dysfunction, and she reported some improvement following pelvic PT. The patient has otherwise remained healthy aside from a history of recurrent sinusitis, spondylolysis, hyperlipidemia, ADHD, and tonsillectomy.

At 29 years of age, the patient delivery a healthy male infant following a relatively uncomplicated pregnancy. Routine prenatal screening labs and fetal ultrasounds completed during the first and second trimesters were unremarkable without evidence of fetal or maternal distress. At 36 weeks of pregnancy, the patient complained of new pruritis of the hands and feet, prompting evaluation of serum bile acids to rule out intrahepatic cholestasis of pregnancy. These were found to be normal, as were AST (24 U/L), ALT (11 U/L), and bilirubin (<0.2 mg/dL). Labor was induced at 37 weeks and 5 days following spontaneous rupture of membranes. On the day of induction, the patient's blood pressure was noted to be mildly elevated (140/80 and 134/91). This prompted an evaluation for pre-eclampsia which was negative, without evidence of proteinuria or hepatic dysfunction (AST 23 U/L, ALT 11 U/L). A vaginal delivery proceeded without complications and her post-partum course was unremarkable.

C4

Patient C4 is an American White male who was born at term via emergency caesarean section due to transverse positioning. The neonatal period was uncomplicated aside from a brief NICU stay due to transient tachypnea of the newborn. His early motor and language milestones were mildly delayed, and he was referred for developmental services due to hypotonia. He was otherwise healthy until about 10 months of age, when he developed several weeks of fever, cough, and irritability that did not improve following a course of outpatient antibiotics. His pediatrician subsequently noted abdominal distension, a palpable spleen, and severe anemia (hemoglobin 6 g/dL), which prompted hospitalization for further workup. In addition to anemia, laboratory studies were notable for elevated transaminases (AST 417 U/L, ALT 86 U/L) and GGT (188 U/L), low albumin (2.7 g/dL), elevated total bilirubin (5.0 mg/dL) and coagulopathy (INR 1.8). At that time, he was also found to have a PICC-line associated DVT. An abdominal ultrasound and CT scan showed marked hepatomegaly with splenomegaly, mild ascites, varices, an enlarged gastric vein, recanalization of the umbilical vein, and an enlarged, echogenic pancreas. Cardiomegaly was also noted, although an echocardiogram showed normal biventricular systolic function at that time. CK was normal at 83 U/L.

The patient was diagnosed with GSD IV at 11 months of age after a liver biopsy showed micronodular cirrhosis and characteristic amylopectin-like inclusions in hepatocytes and macrophages. Genetic testing confirmed the presence of biallelic pathogenic variants in the *GBE1* gene (Table 1). Over the following three months, he was hospitalized multiple times for infection including an enterococcal UTI, adenovirus URI, and *S pneumoniae* bacteremia. His liver function continued to worsen, with increasingly severe hyperbilirubinemia (23.6 mg/dL), coagulopathy (INR 3.0) and thrombocytopenia (94,000) at 1.1 years of age. An echocardiogram showed an interval increase in the size of the left ventricle, although biventricular systolic function was preserved at that time. A cardiac biopsy at 1.1 years of age showed large, PAS-D positive cytoplasmic deposits in cardiomyocytes, in addition to some degree of myocyte disorganization, increased myocyte size, and variability in nuclei.

The patient underwent living donor liver transplantation (maternal donor) at 1.2 years of age. Post-transplant laboratory studies at 1.4 years of age demonstrated adequate hepatic graft function (total bilirubin 0.2 mg/dL, albumin 3.7 g/dL, INR 1.2). His post-transplant course was complicated by CMV viremia, EBV viremia, and a central line-associated subclavian thrombus. He was discharged at 1.4 years of age but was readmitted less than one week later for respiratory distress in the setting of worsening cardiomegaly (BNP >3000 pg/mL) and pulmonary edema. A repeat echocardiogram showed declining systolic function. At 1.6 years of age, he developed cardiogenic shock requiring deployment of ECMO, after which he was converted to LVAD, followed by BiVAD. He continued to require mechanical cardiac support through his death at 1.7 years of age, which was preceded by a massive MCA stroke.

C5

Patient C5 is a Canadian White male who was the product of an uncomplicated pregnancy and caesarean delivery at 38 weeks of gestation. His early gross motor milestones were mildly delayed, and he was noted to have global hypotonia around 4 months of age. After his pediatrician noted hepatomegaly on a routine exam at 9 months of age, he was found to have elevated transaminases (AST 823 U/L, ALT 360 U/L) and GGT (240 U/L) with normal platelets, bilirubin, and INR. An abdominal ultrasound showed an enlarged, hyperechoic liver and splenomegaly. These findings prompted a liver biopsy at 12 months of age, which showed micronodular cirrhosis and characteristic PAS-D inclusions. The diagnosis of GSD IV was confirmed via identification of biallelic pathogenic variants in the *GBE1* gene (Table 1). Although there was no clinical evidence of cardiac involvement (based on echocardiogram, cardiac catheterization, and ECG), a biopsy of the myocardium at 15 months of age showed PAS-D positive deposits in approximately 5% of cardiomyocytes. A skeletal muscle biopsy of the left gracilis was obtained concurrently and was notable only for a predominance of type I fibers with selective atrophy of type 2 fibers. No abnormal polyglucosan inclusions were identified and the glycogen distribution was normal.

The patient underwent living donor liver transplantation at 2.4 years of age. His uncle was the donor. The explanted liver was notable for significant architectural distortion due to a mixture of micronodular and macronodular cirrhosis. His immediate post-transplant course was complicated by an episode of rotavirus hepatitis. He recovered from this infection but continued to exhibit mild elevations of AST, ALT, and GGT over the following months. These persistent laboratory abnormalities prompted a biopsy of the transplanted liver at 2.6 years of age, which was essentially normal without evidence of rejection. Shortly thereafter, he was hospitalized for his first episode of biliary duct stenosis and cholangitis at 2.8 years of age, for which he required biliary stenting and a multi-week course of intravenous antibiotics. Over the following years, he continued to suffer from recurrent cholangitis, including episodes at the ages of 3, 4, 10, 11 and, most recently, 13 years of age. During that time, he also developed multiple PICC line-associated thromboses. A liver biopsy was repeated at 7.7 years of age, which showed evidence of mild cellular rejection but no other gross abnormalities. As of his last available laboratory evaluation at 13.8 years of age, he remained clinically stable with mildly elevated GGT (40 U/L), normal transaminases (ALT 23 U/L, AST 34 U/L), and intact synthetic function (albumin 3.8 g/dL).

The patient's clinical course has otherwise been notable for a progressive decline in walking endurance and decreased exercise tolerance compared to peers. By 10 years of age, he reported needing to rest after just 15 minutes of walking, after having previously been able to tolerate more than an hour of uninterrupted activity. He also reported daily episodes of tripping, intermittent paresthesia in his calves and heels, and longstanding post-void urinary retention. A physiatry evaluation at 9.7 years of age was notable for proximal lower extremity weakness, particularly in the gluteal extensor muscles. CT imaging of the brain at 13.3 years of age was unremarkable aside from bilateral, hyperdense basal ganglia lesions of unclear significance. On MRI, these appeared as areas of slightly increased T1 and decreased T2 signal that were hypointense on SWI. Serial echocardiograms and ECGs have consistently showed normal cardiac morphology and function, most recently at 13.1 years of age. At 13.3 years of age, he underwent an EGD and colonoscopy for chronic epigastric pain of uncertain etiology. Although no gross macroscopic abnormalities were identified, histopathological analysis revealed an eosinophil-rich inflammatory infiltrate in the gastric antrum. Histopathology findings from the esophagus, duodenum, ileum, colon, and rectum were unremarkable.

C6

Patient C6 is an American male of Polish, English, and German descent who was the product of an uncomplicated pregnancy and a term vaginal delivery. Around four to six months of age, he exhibited delayed gross motor milestones, failure to thrive, and abdominal distension. He was diagnosed with GSD IV at 7 months of age after a biopsy revealed absent GBE activity in the liver. The histopathology report from this original liver biopsy is unavailable and *GBE1* genetic testing was not performed.

The patient underwent living donor liver transplantation (maternal donor) at 11 months of age. His early post-transplant course was complicated by EBV viremia and a prolonged requirement for opioids, necessitating the use of clonidine for withdrawal symptoms. Following LT, his hepatic function remained stable aside from a transient, mild increase in bilirubin (1.4 mg/dL) and transaminases (AST 226 U/L, ALT 350 U/L) in the setting of a viral illness at 10.3 years of age. Serial abdominal imaging studies after LT (most recently at 12.2 years of age) have been unremarkable aside from persistent splenomegaly. The patient's transaminases, bilirubin, platelets, and albumin were all normal as of his most recent laboratory evaluation at 13.1 years of age. His cardiac function has been evaluated via serial echocardiograms and ECGs, which were largely unremarkable until 11.4 years of age, when an echocardiogram showed new tricuspid regurgitation, impaired RV relaxation, and mild RV dilation, with preserved biventricular systolic function and normal LV size.

In addition to delayed gross motor milestones, the patient's neuromuscular course was most notable for gradual-onset chronic fatigue and musculoskeletal pain involving his shoulders, elbows, knees, hands, and back. The patient also complained of chronic neck pain, although this is possibly attributable to his cervical spine fusion in the setting of suspected chronic recurrent multifocal osteomyelitis (CRMO), as detailed below. By 10 years of age, he was intermittently using a wheelchair due to leg pain and fatigue. These symptoms became worse in the setting of an unexplained illness at 11.2 years of age, characterized by several weeks of recurrent fevers and severe, diffuse musculoskeletal pain. The patient underwent a thorough inpatient evaluation including full-body MRI (negative for osteomyelitis or arthritis), PET scan (negative for PTLD or other malignancy), rheumatological evaluation, and an extensive infectious disease workup (including cultures, viral panels, lumbar puncture and an NGS-based pathogen detection test) that was positive for rhinovirus but was otherwise unrevealing. A brain MRI showed patchy, bilateral subcortical T2 white matter hyperintensities. The patient's fevers ultimately resolved but he continued to report severe pain and muscle fatigue that were much worse compared to his prior baseline. These symptoms interfered with his participation in school and recreational activities, and at 11.6 years of age, he reported that he could not walk for more than 20 minutes without needing to rest or use a wheelchair. As of his most recent evaluation at 13.8 years, he continued to report arthralgias, myalgias, and gait disturbance.

In addition to the above, the patient's clinical course has been complicated by several other chronic issues. In the years following LT, he developed recurrent pneumonias necessitating multiple hospitalizations, one of which was complicated by bilateral parapneumonic effusions requiring treatment with VATS. He also developed multiple DVTs (some of which were central line-associated), which prompted a thrombophilia workup at 6.8 years of age that was negative. At 7.3 years of age, he began complaining of severe, chronic neck pain. Initial imaging studies were non-diagnostic until an MRI at 7.5 years of age showed bone and soft tissue enhancement with atlantoaxial dislocation. These findings were concerning for bacterial osteomyelitis, although cultures of the biopsied lesion were negative. He subsequently developed a large elbow effusion, and cultures of the aspirate were again negative. He was diagnosed with CRMO and underwent cervical spinal fusion surgery at 7.8 years of age. The patient has also been hospitalized multiple times for pancreatitis (9.5, 11.4, and 12.2 years of age) and for multiple febrile illnesses of unclear etiology (presumed viral), often with marked elevation of serum inflammatory markers (CRP, ESR).

C7

Patient C7 is an American White male who was previously described by McConkie-Rosell et al. (Patient D) in a 1996 case series of patients with the so-called "non-progressive hepatic" subtype of GSD IV. The current study reports approximately 20 years of additional follow-up data from this patient.

Patient C7 was the product of a full-term, uncomplicated pregnancy and delivery. He achieved his early developmental milestones on time and had no major illnesses during the first year of life. Thereafter, he began to show signs of abnormal growth velocity. Although his length tracked along the 25th percentile during the first year of life, he had dropped to the 5th percentile by 21 months of age and was well below the 5th percentile by three years of age. This prompted a workup for growth failure which revealed elevated transaminases (ALT 100 U/L, AST 149 U/L) and hepatomegaly. A liver ultrasound showed normal hepatic echogenicity with no lesions. Over the following year, his transaminases continued to rise (max ALT 299 U/L; max AST 467 U/L) although his bilirubin, albumin, platelets, and INR remained normal. An inpatient evaluation showed no definite evidence of hypoglycemia, although his fasting blood sugars were noted to be borderline-low (around 65 mg/dL) on several occasions, and his parents reported that he was often "ravenous" upon awakening in the morning. He underwent a liver biopsy at 4.1 years of age which showed small regenerative nodules surrounded by fibrous bands, in addition to enlarged hepatocytes with PAS positive deposits that were largely sensitive to diastase. The diagnosis of GSD IV was confirmed via identification of decreased fibroblast GBE activity at 4.5 years of age. There was no evidence of neuromuscular involvement and his CK was normal.

Since his case was last reported in the published literature at five years of age, patient C7 has exhibited significant improvement in his hepatic and growth parameters. He continued to grow at or below the 5th percentile through 7.5 years of age, which prompted an endocrine workup that revealed delayed bone age, partial growth hormone deficiency, and a small anterior pituitary gland with no mass lesions on MRI. He was started on somatropin, resulting in normalization of his growth. His liver size and transaminases progressively declined and were normal by age ten. An abdominal ultrasound at 16 years of age was notable only for mild splenomegaly, with normal hepatic echotexture and normal Doppler flow. During adolescence, he was reported to have mildly decreased exercise tolerance compared to peers, although he remained physically active and participated in sports. His CK remained normal.

Patient C7 remained stable through his most recent available follow-up at 25.3 years of age. After his AST was noted to be mildly elevated (68 U/L) at 24.8 years of age, an abdominal MRI was obtained that was again notable only for splenomegaly. The appearance of the liver was normal aside from a benign appearing, septated cyst. His transaminases were normal when re-checked two months later. An echocardiogram at 25.0 years of age showed a normal ejection fraction of 55%.

C8

Patient C8 is an American White male who presented at 1.5 years of age with increased abdominal girth and hepatosplenomegaly on examination. These findings prompted a liver and skin biopsy that confirmed the diagnosis of GSD IV. His growth was abnormal, with length and weight both below the 5th percentile. He did not develop ascites, bleeding varices, or hepatic encephalopathy. An echocardiogram at 1.6 years of age was unremarkable.

The patient underwent LT at 2.7 years of age with no major complications. The explanted liver was noted to be cirrhotic and enlarged. Over the following years, laboratory studies were notable for intermittent, mild elevations in transaminases (AST and ALT remaining <100 U/L) with preserved synthetic function of the grafted liver. A liver biopsy at 13.1 years of age showed minimal portal inflammatory infiltrate without evidence of rejection or infection. As of his last hepatic laboratory evaluation at 27.6 years of age, he had normal transaminases (AST 20, ALT 18), total bilirubin (0.4 mg/dL), and albumin (4.6 g/dL).

Although his pre-transplant echocardiogram was normal, the patient ultimately developed severe cardiac dysfunction leading to multiple complications during the third decade of life. During late adolescence, he developed atrial fibrillation that led to a stroke around 21 years of age, after which a cardiac ablation was performed. A diagnostic cardiac catheterization at 24.2 years of age showed moderate to severe pulmonary hypertension. During the procedure, an endomyocardial biopsy was obtained that showed moderate to marked myocyte hypertrophy with PAS-positive inclusions (partially resistant to diastase digestion) in a minority of cardiomyocytes. An echocardiogram at that time was notable for a mildly enlarged left ventricle (LV) with a low ejection fraction (EF) of 20-25%, in addition to a restrictive filling pattern consistent with diastolic dysfunction. The right ventricle was also enlarged with moderate to severe systolic dysfunction. A subsequent echocardiogram at 24.4 years of age showed similar findings in addition to a new LV thrombus that developed despite use of anti-coagulation. Placement of an automatic implantable cardioverter defibrillator (AICD) was attempted at 25.2 years of age, although his post-operative course was complicated by poor wound healing and a pocket hematoma, necessitating removal of the device a month after placement. The procedure was subsequently re-attempted and an AICD was successfully placed at 25.6 years of age. However, he was noted during a pre-operative workup to have an occlusive thrombus in the left subclavian vein. His cardiac function has remained stable as of latest follow up at 32.2 years, with EF 25-30% on echocardiogram and normal sinus rhythm on ECG at 31.7 years of age.

C9

Patient C9 is a German female whose clinical course was initially described through 3.4 years of age by Derks et al. (Patient 10). The current study reports approximately three years of additional follow-up data from this patient.

Patient C9 was delivered via caesarean section at 38 weeks of gestation, following a pregnancy complicated only by decreased fetal movements. At birth, she was noted to have arthrogryposis, global hypotonia, talipes equinovarus, and scoliosis. Laboratory tests revealed elevated CK. These findings prompted exome sequencing at 3 months of age, which identified biallelic *GBE1* mutations (Table 1). The diagnosis of GSD IV was confirmed via measurement of decreased leukocyte and erythrocyte GBE activity. Biopsy was not performed.

Although the patient's transaminases were normal when assessed during the first nine months of life, ALT was noted to be mildly elevated (37 U/L) at 12 months of age, followed by a further increase to 64 U/L at 20 months of age. ALT subsequently decreased and all measurements from 2.2 years through her most recent laboratory evaluation at 6.2 years of age remained within the normal range. Abdominal ultrasounds at 6 months and 2.8 years of age showed no hepatic abnormalities, although liver echogenicity was noted to be slightly increased at 4.8 years of age. Despite her otherwise normal liver function – without evidence of portal hypertension or synthetic dysfunction – she was found to have elevated fasting ketones and hypoglycemia, requiring initiation of a high-protein diet with uncooked cornstarch supplementation and a late-evening meal beginning around 2.8 years of age. These dietary modifications resulted in an improvement in morning ketone levels, from typical values of >1.0 mmol/L previously to around 0.3 mmol/L currently. However, she remains susceptible to ketosis during periods of illness, and most recently required hospitalization at 5.8 years of age due to poor oral intake in the setting of tonsillitis.

As of the patient's most recent neuromuscular evaluation at 5.8 years of age, she was noted to exhibit hypotonia, scoliosis, hyperlordosis, and bilateral contractures that were most prominent in the distal extremities. At that time, she continued to use a wheelchair for mobility but had made significant progress towards rising to stand without assistance. Her speech was age-appropriate. Cardiac evaluations, including her most recent available echocardiogram at 5.3 years of age, have been unremarkable aside from a patent foramen ovale.

C10

Patient C10 is an American White male of Irish and Cherokee descent who was the product of an uncomplicated pregnancy and term delivery. His neonatal period was notable only for mild hyperbilirubinemia and ankyloglossia requiring a frenulectomy. His early development and medical history were unremarkable until 1.5 years of age, when he was noted to have hepatomegaly on a routine examination. Laboratory studies at that time were notable for elevated AST (705 U/L), ALT (494 U/L) and GGT (234 U/L). His albumin, bilirubin, and platelets were all normal, although he was found to have a borderline elevated PT (14.9s; normal: <14.6s) and INR (1.2; normal: <1.2). An ultrasound at 1.6 years of age showed hepatosplenomegaly with a mottled hepatic echotexture. These findings prompted a liver biopsy at 1.7 years of age, which showed extensive bridging fibrosis and a focal regenerative nodule suspicious for cirrhosis. The diagnosis of GSD IV was confirmed via identification of biallelic *GBE1* variants (Table 1).

By 3.4 years of age, the patient's transaminases had begun to improve (AST 289 U/L, ALT 208 U/L), although he exhibited a concurrent worsening of his INR (1.3) and platelet count (89,000). An abdominal ultrasound with shear wave elastography (SWE) at 4.5 years of age showed hepatosplenomegaly with a normal hepatic echotexture and a normal shear wave velocity (SWV) of 1.0 m/s. However, SWV was mildly abnormal (1.65 m/s, corresponding to the F1 fibrosis range) on a subsequent SWE study at 5.0 years of age. He has not exhibited low albumin or elevated bilirubin. EGD evaluations at 4.8 and 5.2 years of age showed no evidence of esophageal varices, although he was incidentally found to have eosinophilic esophagitis and focal lamina propria fibrosis in the gastric antrum. He was stable as of his last clinical evaluation at 5.2 years of age and has not exhibited any evidence of neuromuscular involvement (normal CK and neurological examination) or cardiac involvement (normal echocardiogram and ECG) to date.

C11

Patient C11 is an American male of Israeli and Ashkenazi Jewish descent whose clinical course was previously described through 3.6 years of age by Derks et al. (Patient 9). The current study reports approximately 2.5 years of additional follow-up data from this patient. He is the younger brother of C12.

Patient C11 patient was born at term following an uncomplicated pregnancy. After his older brother was diagnosed with GSD IV, patient C11 underwent genetic testing at 8 months of age that confirmed the presence of biallelic *GBE1* mutations (Table 1). Neither a liver biopsy nor enzymology was performed. Laboratory studies revealed elevated transaminases, which peaked at ten months of age (AST 236 U/L, ALT 245 U/L) and decreased thereafter, returning to the normal range by three years of age. His transaminases remained normal (ALT 25 U/L, AST 28 U/L) as of his most recent laboratory evaluation at 6.1 years of age. Hepatic echotexture was reported as normal on all available ultrasonography studies (at 1.5, 3.4, and 4.3 years of age). Although the spleen was noted to be slightly enlarged on the 4.3 year ultrasound, there was no evidence of splenomegaly on his most recent abdominal MRI at 5.5 years of age. His platelets, bilirubin, and synthetic function have consistently remained normal. Despite his otherwise intact liver function, he has had multiple hospitalizations for hypoglycemia due to decreased oral intake in the setting of gastroenteritis or other febrile illnesses. He follows a high-protein diet with UCCS supplementation for maintenance of normoglycemia.

Although the patient acquired his early gross motor milestones on time, his parents have since noted that he tends to fatigue more readily than peers. He has also been reported to stumble or fall frequently, and he often complains of painful muscle cramps in his lower extremities. He can ambulate independently over short distances but has a wheelchair available for use as needed. CK has been measured at multiple time points (beginning at 9 months of age) and has never been elevated. During a comprehensive physical therapy evaluation at 5.2 years of age, he was observed to exhibit lumbar lordosis, circumduction gait, excess pelvic rotation, and increased reliance on upper extremity movement for momentum during walking. Additionally, he was incidentally noted to have diffuse skeletal muscle atrophy on a cardiac MRI at 5.5 years. A brain MRI showed a small focus of hyperintensity on T2/FLAIR in the left centrum semiovale, felt to possibly represent minimal nonspecific gliosis. With respect to his cardiac function, the same MRI showed normal biventricular size and borderline global systolic function (LVEF 50%). However, the latter finding was ultimately attributed to the effects of anesthesia during the MRI, after a subsequent echocardiogram at 6.0 years of age showed a normal LVEF of 70%. His prior ECGs and echocardiograms have also been normal aside from a finding of LV trabeculations.

Patient C11 has also been followed for chronic issues with nausea, vomiting, flushing, rapid weight gain, and sleep disturbances which began at 2 years of age. An extensive endocrine and gastroenterology workup – including a gastric emptying study, upper GI series, stool studies, EGD, and colonoscopy – was non-diagnostic. Notably, however, histopathological evaluation of biopsy samples from the stomach and colon showed an increased number of lamina propria histiocytes that were highlighted by PAS and PAS-D stains. Per the associated pathology report, the histiocytes were benign in appearance and were not felt to reflect an infiltrative process, but rather, suggested a potential role of the histiocytes in the “clean-up” of glycogen deposits associated with the patient’s known GSD. An ill-defined rectal granuloma was also noted. The patient ultimately required placement of a gastrostomy tube for recurrent vomiting. He continued to report frequent nausea with intermittent emesis as of his most recent evaluation at 6.1 years of age.

C12

Patient C12 is an American male of Israeli and Ashkenazi Jewish descent whose clinical course was previously described by Derks et al. (Patient 8) through 6.3 years of age. The current study presents approximately 2.5 years of additional follow-up data from this patient. He is the older brother of Patient C11.

Patient C12 was born at term following an uncomplicated pregnancy and delivery. There were no reported complications during the neonatal period. Around 2.5 years of age, he was noted to be more fatigable than his peers, which prompted an evaluation that revealed elevated transaminases (AST 296 U/L, ALT 281 U/L) and hepatomegaly. A liver biopsy at 3.1 years of age showed bridging fibrosis with regenerative nodule formation, in addition to PAS-positive, partially diastase-resistant deposits in histiocytes and hepatocytes. The diagnosis of GSD IV was confirmed via identification of biallelic *GBE1* mutations (Table 1) at 3.2 years of age. Enzymology studies were not performed. At four years of age, the patient had an episode of nocturnal tremulousness witnessed by his parents. Thereafter, fingerstick measurements showed that he was having intermittent episodes of hypoglycemia and elevated ketones (up to 2.9 mmol/L). He was started on a high-protein diet with UCCS supplementation. These dietary interventions were largely successful in maintaining euglycemia, although he nonetheless was hospitalized on multiple occasions for hypoglycemia during periods of decreased oral intake. Over time, the patient's hepatomegaly improved, and his serum transaminases normalized by five years of age. His hepatic echotexture was reported to be abnormal on ultrasounds at 4.3, 6.2, and 7.1 years of age. However, his most recent abdominal imaging study (MRI with elastography) at 8.4 years of age showed a normal, homogeneous liver appearance with a mean hepatic stiffness of 1.74 kPa (normal <2.5 kPa). The spleen was noted to be slightly enlarged on the patient's 7.1 year ultrasound and was reported as "top-normal" on his subsequent 8.4y MRI. Imaging studies were otherwise unremarkable. His most recent laboratory evaluation at 8.9 years of age showed normal transaminases (AST 24 U/L, ALT 26 U/L) and normal GGT (13 U/L) without laboratory evidence of portal hypertension or synthetic dysfunction.

Developmentally, patient C12 acquired his early motor milestones on time and was able to walk by 15 months of age. As noted previously, however, he showed signs of decreased muscle endurance beginning at around 2.5 years of age, which prompted the evaluation that led to the diagnosis of GSD IV. Since that time, the patient has continued to exhibit impaired exercise tolerance. A physical therapy evaluation at 8.0 years of age showed scapular winging, lordosis, kyphosis, rib flaring, pes planus, and lower extremity weakness during functional movements. Notably, however, a biopsy of the quadriceps muscle at 8.3 years of age showed no evidence of abnormal polyglucosan inclusions, with normal glycogen content and morphology on electron microscopy. Muscle fiber size and type distribution were also normal, and there was no evidence of inflammation, necrosis, or fibrosis. As of his most recent evaluation at 8.9 years of age, the patient continued to report musculoskeletal pain with exertion and increased fatigability compared to peers, requiring frequent rest breaks and intermittent use of a wheelchair. He also complained of muscle fatigue in his jaw while chewing and in his postural muscles while sitting upright at school. His most recent echocardiogram at 8.8 years of age showed a trabeculated LV wall with otherwise normal cardiac morphology and function.

The patient's clinical course has otherwise been notable for adenotonsillectomy at 4.3 years of age; turbinate hypertrophy requiring resection and a microcystic lymphatic malformation of the tongue requiring excision, performed simultaneously at 8.3 years of age; and premature pubarche with advanced bone age at 8.8 years of age.

C13

Patient C13 is a Canadian male of Mennonite descent with European and Russian ancestry. His birth and family history are unmarkable; the patient has a left solitary kidney as a result of right renal agenesis/dysplasia. He was diagnosed with GSD IV at 2.9 years old by whole exome sequencing (Table 1) after presenting with elevated hepatic transaminases (AST 270 U/L and ALT 224 U/L) and enlarged liver at 2.4 years old. At age 2.7 years, the patient had a liver biopsy which revealed cirrhotic changes with glycogen inclusions consistent with GSD IV. Abdominal imaging (ultrasound) revealed that the liver was normal in size at 2.3 years, had coarsened echotexture at 2.8 years, had subtle nodularity (particularly in the left lobe) at 3.2 years, remained at a normal size with thickened portal vein walls at 3.6 years, and coarsened echotexture at 4.6 years. The spleen has not been noted to be enlarged. At 3.1 years, neurological exam revealed that tone was slightly hypotonic peripherally with normal muscle bulk. On gait exam, the patient was tired while walking with progressive circumduction of his legs and slowing of pace; he had difficulty climbing stairs and had to recover after each step and was able to rise from sitting with some difficulty. Proximal weakness was noted but muscle strength was not able to be tested due to age and compliance. He was referred to physical therapy and by 4.5 years, the follow up neurological exam was unremarkable with normal gait, coordination, balance, and strength. The only noted weakness was when walking downstairs. A 16-hour fasting test at 4.0 years was unremarkable and since 4.1 years of age, the patient has followed a low carbohydrate diet with avoidance of simple sugars. The patient was 5.1 years at his most recent visit and had no evidence of cardiac involvement. He is considered to be stable and last blood chemistries at 4.9 years revealed elevated (but decreased) transaminases (AST 97 U/L, ALT 98 U/L); total protein, albumin, bilirubin, and PT and INR have remained within normal limits.

C16

Patient C16 is an Indian male who had an unremarkable birth and family history. He initially presented with upper abdominal distension at 8 months which progressed and by 16 months, the patient had yellow discoloration of the eyes and urine. This further progressed to ascites at 18 months and prompted a liver biopsy at 20 months which revealed F4 fibrosis (Metavir score) and enclosed parenchymal nodules indicative of cirrhosis, as well as PAS-positive diastase-resistant cytoplasmic inclusions. Whole exome sequencing confirmed biallelic variants in *GBE1* (Table 1). Abdominal imaging (ultrasound and CT scan) revealed an enlarged liver (11 cm span) with coarse echotexture, an enlarged spleen (10 cm span), and mild free fluid in the abdomen at 16 months. At 20 months, the liver was enlarged (9.5 cm span) with hypertrophied left and caudate lobes and cirrhotic architecture. At 24 months, liver was enlarged at 9.5 cm span and spleen was enlarged at 10 cm span; periportal edema was noted along with a dilated splenoportal axis and multiple abdominal collaterals. Additionally, Fibroscan imaging at 20 months indicated liver stiffness of 66 K Pa. Upper gastrointestinal endoscopy indicated small to large high-risk esophageal varices. At 20 months of age, the patient had elevated AST (526 U/L), ALT (172 U/L), ALP (210 U/L), GGT (89 U/L) total bilirubin (3.76 mg/dL), conjugated bilirubin (1.6 mg/dL), PT (24 s), and INR (2.2), and reduced albumin (2.5 g/dl); by 24 months of age, the patient's status worsened and he had elevated AST (952 U/L), ALT (187 U/L), 64 (U/L), total bilirubin (12.1 mg/dL), conjugated bilirubin (6.1 mg/dL), PT (41.5 s), and INR (3.84), and reduced total protein (4.3 g/dL) and albumin (2.6 g/dL). The patient did not ever present with ketonuria or history of symptomatic hypoglycemia, but had fasting hypoglycemia with end stage liver disease. Height and weight were consistently below average (76 cm at 20 and 24 months, 10.1 kg at 20 months, 8.4 kg at 24 months). The patient's PELD score was 21 at 20 months of age and hepatic encephalopathy grade progressed from 1 at 20 months to 3 at 24 months. He ultimately died while on the liver transplant waiting list at 24 months from pulmonary hemorrhage with disseminated intravascular coagulation (DIC) with hemoperitoneum and septic shock related to advanced end stage liver disease. At time of death, the patient also had persistent coagulopathy-related gastrointestinal bleed, acute kidney injury and hyponatremia, and decompensated chronic liver disease (jaundice, ascites, hepatic encephalopathy grade III) with portal hypertension. The patient had no history of cardiac (normal echocardiogram and electrocardiogram), muscle, or nervous system involvement. The patient maintained a high protein, high calorie diet consistent with management of decompensated chronic liver disease along with vitamin D, calcium, trace element, and multivitamin supplementation from 21 months of age until death.

C18

Patient C18 is a Nicaraguan Latina female who was born via caesarean section at 40 weeks of gestation following an uncomplicated pregnancy. She achieved her early developmental milestones on time and began walking around 12 months of age. However, around 2 years of age, she was noted to have an abnormal gait, hyperlordosis, and significant fatigability. An EMG was reportedly normal at that time, and WES at 3.9 years of age was non-diagnostic. She was otherwise healthy until 4.3 years of age, when she was hospitalized for acute abdominal pain and recurrent emesis with severe gastric distension on imaging. An EGD showed extensive congestion and ulceration of the stomach with severe pyloric edema that obscured the gastric outlet, necessitating approximately 1 week of TPN. During that admission, her transaminases were reportedly normal. After discharge, she remained well until 4.5 years of age, when she was again admitted for severe abdominal pain with new hepatomegaly, elevated transaminases (AST 1998 U/L, ALT 446 U/L), elevated GGT (1017 U/L), elevated lipase (462 U/L), and ultrasound evidence of pancreatic and hepatic inflammation. Subsequent imaging studies, including an MRCP and CT scan at 4.6 years of age, showed mild periportal edema with an otherwise normal appearance of the liver, spleen, and pancreas, although her transaminases (AST 1978 U/L, ALT 346 U/L) and lipase (1180 U/L) remained elevated. A liver biopsy at 4.6 years of age showed PAS-positive cytoplasmic inclusions that were partially sensitive to diastase, in addition to "portal, periportal and bridging fibrosis with nodularity and mixed inflammatory infiltrates most suggestive of early cirrhosis." These findings prompted enzymology studies which confirmed deficient GBE activity and elevated glycogen content in the liver. Re-analysis of her previously non-diagnostic WES also identified biallelic *GBE1* mutations (Table 1).

The patient has since been followed through 8.3 years of age. Her transaminases declined over time and were normal from 6.9 years of age and onward. Aside from prominent veins on the surface of her abdomen, she has not shown signs of portal hypertension such as splenomegaly, varices, or thrombocytopenia. She had several borderline-elevated INR values from four to six years of age, although her synthetic function otherwise remained normal (albumin > 3.8 g/dL). She has not had clinical or laboratory evidence of hypoglycemia. Hepatic elastography measurements based on acoustic radiation force imaging (ARFI) at 5.3 and 7.4 years of age were consistent with normal hepatic stiffness based on shear wave speeds of 1.25 m/s and 1.17 m/s, respectively. However, an MR elastography study at 6.3 years of age showed a borderline-elevated liver stiffness of 2.81 kPa (normal <2.75 kPa). Cardiac evaluations, including her most recent ECG and echocardiogram at 8.3 years of age, have been normal. Her neuromuscular course has been notable for ongoing gross motor difficulties, and her parents have reported that she continues to be clumsier and slower than peers with frequent falls. Additionally, her abdominal and paraspinal musculature have been noted to be atrophic on CT imaging. A skeletal muscle biopsy was reportedly unremarkable aside from non-specific, mild type 1 fiber predominance. All available CK values have been normal.

Her clinical course has otherwise been complicated only by gastric ulceration of unclear etiology. As noted previously, she initially presented with pyloric obstruction in the setting of severe edema and mucosal ulceration at 4.3 years of age. The ulcerations were reported to have healed on a subsequent EGD performed the following year, although the mucosa showed evidence of scarring at that time. At 5.7 years of age, she presented with another episode of acute abdominal pain and was found to have a small gastric perforation, reportedly at the site of the healed ulcerations. Since that time, she has been stable on esomeprazole therapy without further episodes of abdominal pain. Her gastric biopsies were reviewed by multiple specialists without identification of a clear etiology.

C26

Patient C26 is an American male of unspecified Hispanic descent and brother of C32. He was born at 42 weeks of gestation following an uncomplicated pregnancy, with appropriate growth parameters for his gestational age. There were no complications reported during labor or delivery, and he was discharged home with his mother. During infancy, the patient exhibited delayed motor milestones. However, he achieved independent walking by two years of age. He also presented with speech delays and feeding difficulties. During infancy he had several hospital admissions related to recurrent pneumonia episodes. At the age of 1 year, he was evaluated by a pulmonologist due to his recurrent respiratory issues. Physical examination at that time revealed hepatomegaly, prompting further investigation. Liver enzyme tests were reportedly elevated, with AST and ALT levels both in the 300 range. A subsequent liver biopsy at age 2.0 years revealed portal fibrosis with thin fibrous strands extending into the lobules; there is no bridging or definite nodularity in the tissue received for histologic review (small sample). Pathology also noted enlarged hepatocytes with cytoplasmic glycogen accumulation and central hepatocellular aggregates of glycogen mixed with fibrillary material, as evident on electron microscopy. There was portal and lobular inflammation which was predominantly lymphocytic with few neutrophils and eosinophils. PAS with diastase staining demonstrated PAS-positive diastase-resistant aggregates. Additional findings included focal lipid droplets, lysosomes, and a redistribution of cellular organelles, such as mitochondria, to the cell periphery. A GSD IV diagnosis was suspected and enzyme testing was sent for further confirmation (Table 1). Genetic testing revealed compound heterozygous variants in the *GBE1* gene (Table 1) confirming the diagnosis. At 2.3 years of age, the patient underwent liver transplantation. Liver explant pathology demonstrated extensive diffuse fibrosis associated with portal inflammation (mixed with lymphocytes predominating). There were no definite regenerative nodules, but a suggestion of micronodularity was created by the diffuse fibrosis. His post-operative course was complicated by posterior reversible encephalopathy syndrome (PRES), presumably associated with tacrolimus use. He also experienced persistent hypertension and episodes of acute cellular rejection. He was stable as of his last evaluation at 19.4 years of age. It is unknown if he has any cardiac or neuromuscular involvement.

C32

Patient C32 was an American female of unspecified Hispanic descent, diagnosed with GSD IV at 3 weeks of age due to a family history of the disease in her brother (C26). She was born at term following an uncomplicated pregnancy. The initial diagnosis was made through skin biopsy, which demonstrated deficient glycogen branching enzyme activity (Table 1). Subsequent genetic testing revealed compound heterozygous variants in the *GBE1* gene (Table 1). During her early development, she manifested symptoms of hypotonia and gross motor delay. She also developed hepatomegaly and elevated liver enzymes between 6 months to 1 year of age. At 6 months of age, her ALT and AST levels were 139 U/L and 121 U/L, respectively. By 11 months of age, these values further increased to ALT 230 U/L and AST 306 U/L. At 3.5 years of age, she received a liver transplant. Her post-transplant course was complicated by chronic rejection, cirrhosis, portal hypertension, and biliary stenosis. Subsequent hospital admissions were necessitated due to chronic rejection and progressive liver failure. At 13.9 years of age, she was admitted to the Pediatric Intensive Care Unit (PICU) for respiratory distress and hypoxemia. Her PICU course was complicated by rapid respiratory decompensation, requiring intubation, progression to acute respiratory distress syndrome (ARDS), potential pulmonary hemorrhage, severe catecholamine-resistant shock, cardiac dysfunction, liver failure, coagulopathy, acute kidney injury (AKI), and severe metabolic acidosis. She was unresponsive to high-dose pressor support, and after evaluation, was deemed not to be an extracorporeal membrane oxygenation (ECMO) candidate. Following discussions with the parents and a decision to redirect care, she rapidly progressed to cardiac arrest and died peacefully at 13.9 years of age.

C36

Patient C36 is an Indian male who was diagnosed with GSD IV at 13 months of age. Family history included sudden death in paternal cousin at 11 months old (details not known). Additionally, the patient's mother reported a history of one intrauterine fetal demise (IUFD) and one spontaneous abortion prior to this patient's gestation. The patient was born at 42 weeks gestation at 4.5 kg and had mild respiratory distress at birth (transient tachypnea) which resolved after 24 hours and did not require ventilation. His medical history was then unremarkable until 10 months of age when he presented with progressive, generalized abdominal distension related to ascites, and then yellow discoloration of the eyes and urine at 12 months of age. The patient had a liver biopsy at 13 months which revealed F4 fibrosis with bridging septae and parenchymal nodules, indicative of cirrhosis. Additionally, the biopsy revealed PAS-positive diastase-resistant cytoplasmic inclusions – consistent with GSD IV. Patient diagnosis was confirmed by whole exome sequencing which revealed biallelic *GBE1* variants (Table 1). Abdominal liver imaging (ultrasound and CT scan) indicated early parenchymal liver disease with findings suggestive of portal hypertension. The liver cranio-caudal span was 10.8 cm and showed subtle, irregular outlines with coarsened echotexture. Left and caudate lobes were hypertrophied. The spleen was enlarged (span 10 cm) with prominent splenoportal axis and normal echotexture. Kidneys and pelvicalyceal systems were unremarkable. Mild free fluid was seen in the peritoneal cavity. Additionally, upper GI endoscopy was suggestive of small low risk esophageal varices and mild portal hypertensive gastropathy. Blood chemistry measurements at 13 months revealed elevated AST (403 U/L), ALT (44 U/L), ALP (281 U/L), GGT (109 U/L), AFP (300 ng/mL), total bilirubin (4.15 mg/dL), conjugated bilirubin (2.2 mg/dL), PT (21.4 s) and INR (1.84), and decreased BUN (1.3 mEq/L). The patient had no history of hypoglycemia or ketonuria. At 13 months of age, he was 78 cm (-0.21 Z score) and 9.53 kg (-0.21 Z score) and his PELD score was 14. He was placed on a high protein, high calorie diet for management of decompensated chronic liver disease. Additionally, he received vitamin D, calcium, trace element, and multivitamin supplementation. The patient received a liver transplant at 15 months and had no reported complications. At that time, he also had a normal echocardiogram and electrocardiogram, and no signs of muscle or nervous system involvement.

C43

Patient C43 is a Brazilian female who was born following a normal pregnancy. Newborn screening and development were normal with no delay or decline. The patient initially presented at 15 days of age with jaundice. Two months later, she developed acholia, and then hepatosplenomegaly at 10 months of age. She underwent a liver biopsy at age 1.4 years which demonstrated PAS-positive staining, ballooning, and micronodular cirrhosis. At that time, hepatosplenomegaly was noted by ultrasound and she was assigned a PELD score of 3. Liver function tests were abnormal with an elevated ALT (101 U/L), AST (74 U/L), GGT (81 U/L), and bilirubin (2.34 mg/dL), alongside a normal albumin (3.9 g/dL). Coagulation markers were also abnormal with elevated PT (18.5 secs) and elevated INR (1.4). At this time, echocardiogram showed light pulmonary hypertension, but a normal ECG. The patient subsequently underwent liver transplantation at 2.3 years. At age 3 years, both ECG and echocardiogram were both normal, and a repeat ultrasound at age 5 years was normal. At 11 years, a repeat liver biopsy was performed that showed a reactive liver. She was most recently seen at age 12.2 years and a third liver biopsy was performed that continued to show reactive liver. Her blood chemistries were largely normal (ALT 48 U/L, AST 39 U/L, bilirubin 0.33 mg/dL, PT 12.8 sec). However, GGT and INR were elevated (GGT 133 U/L, INR 1.23). Liver ultrasound, ECG, and echocardiogram were all normal, and the patient continues to be ambulatory with no evidence of neuromuscular involvement throughout their clinical history.

C44

Patient C44 is a Brazilian female who was initially diagnosed with GSD IV at 9 months of age after presenting with hepatomegaly at 8 months of age. A liver biopsy at 9 months old was consistent with a GSD IV diagnosis and whole exome sequencing revealed two variants in *GBE1* (Table 1). Previous medical history was unremarkable; the patient was born at 38 weeks gestation (3.06 kg) and exhibited jaundice on day 4 which was treated with phototherapy. At 10 months old, she exhibited failure to thrive, hepatosplenomegaly, esophageal varices, upper gastrointestinal bleeding, and the patient's PELD score was 3. At that time, laboratory values indicated elevated AST (384 U/L), ALT (117 U/L), GGT (333 U/L), total and direct bilirubin (1.45 and 1.23 mg/dL, respectively), INR (1.16), and partial thromboplastin time (43.4 s); albumin was within normal limits (3.6 g/L) and platelet count was reduced (87,000). At 14 months of age, the patient received a liver transplant. The patient was 17 months old at her most recent follow up, and laboratory values indicated normal AST (36 U/L), ALT (45 U/L), GGT (14 U/L), total and direct bilirubin (0.18 and 0.07 mg/dL, respectively), albumin (4.1 g/L), INR (1.1), partial thromboplastin time (29.9 s), and platelet count (201,000). The patient has not followed a specific diet and takes a multivitamin. There was no evidence of neuromuscular or cardiac involvement (ECG normal at 10 months) nor a history of hypoglycemia or ketonuria.

C45

Patient C45 is a Native Hawaiian male who was previously described by McConkie-Rosell et al. (Patient B) in a 1996 case series of patients with the so-called “non-progressive hepatic” phenotype of GSD IV. He was then diagnosed with the “adult-onset” neurodegenerative form of the disease (adult polyglucosan body disease, APBD) in his 30s as published in 2017 by Lee et al. (The patient). The current study reports the autopsy liver findings. He died at age 47.8 years from medically assisted death and autopsy samples of the left and right lobe were collected. Minimal PAS-positive diastase-resistant inclusions were observed in the left and right lobe sections. Masson’s trichrome and H&E staining demonstrated bridging fibrosis and steatosis in the right lobe and cirrhosis in the left lobe along with sinusoidal dilatation. A 5x4 cm pedunculated mass was observed on the left lobe; H&E staining of a sample of the mass confirmed that it was a hemangioma which was considered to be benign and likely unrelated to GSD IV.

C61

Patient C61 is an American male from Saudi Arabia. This patient underwent orthotopic liver transplantation at age 3 years. Explant pathology demonstrated micronodular cirrhosis and storage material within hepatocytes consistent with amylopectin (ultrastructural) and staining PAS-positive diastase-resistant. A mild to moderate chronic inflammatory infiltrate was present involving primarily the fibrotic regions with focal small lymphocytic aggregates within the hepatic parenchyma. The patient had molecular genetic testing which confirmed the presence of two variants in *GBE1* (Table 1). He was 19.3 years old at his last reported follow up. It is unknown if he has any cardiac or neuromuscular involvement.

C63

Patient C63 is an American White Hispanic male. He was born following an uncomplicated pregnancy and spontaneous vaginal delivery, and his size and development were appropriate for his gestational age. At 3 months of age, during a hospitalization for cellulitis, he was observed to have persistently elevated liver enzymes, which prompted further evaluation. An abdominal ultrasound revealed hepatomegaly. A subsequent liver biopsy at 4 months of age demonstrated portal fibrosis with focal bridging and hepatocytic glycogenic pseudo-ground glass inclusions in the cytoplasm, positive with PAS staining and consistent with polyglucosan material. Assessment of GBE activity in this tissue confirmed deficiency. Genetic testing revealed two heterozygous pathogenic variants in *GBE1* (Table 1), confirming the diagnosis of GSD IV. At 5 months, an echocardiogram did not show any signs of cardiomyopathy, and there were no discernable neuromuscular manifestations. However, by 10 months, he exhibited severe hepatomegaly, splenomegaly, and ascites, with liver enzymes elevated (AST 600 U/L, ALT 178 U/L, GGT 323 U/L). He had mild icterus (bilirubin 3.7 mg/dL), hypoalbuminemia (albumin 2.9 g/dL), and a stable elevation of INR (1.4-1.6). Laboratory findings were also concerning for microcytic anemia (hemoglobin 7.2 g/dL). Significant feeding difficulties necessitated nasogastric tube placement, and he was diagnosed with global developmental delay. Despite being planned for liver transplantation at 13 months of age, the procedure was not performed due to the unavailability of a suitable donor graft. His condition deteriorated with the development of respiratory failure secondary to rhinovirus infection, leading to intubation, and subsequent progression to liver failure, pulmonary hemorrhage, acute kidney failure requiring continuous renal replacement therapy (CRRT), and septic shock. Despite maximal support with mechanical ventilation, pressors, and CRRT, he developed refractory multiorgan failure. His course was complicated by bilateral pneumothoraxes, necessitating the placement of two chest tubes. His oxygenation worsened despite maximal ventilatory support, and he eventually passed away at 13 months of age.

C66

Patent C66 is an American male born at 36 weeks of gestation. Ethnic background was not available. Detailed data from his birth and the first year of life is limited. According to previous medical records, the patient had been exhibiting gross motor delays until his 9-month well-child visit. During this visit, his primary care physician detected hepatomegaly and elevated ALT levels. Given these abnormal findings, the child was referred for a liver biopsy at 11 months of age. The biopsy histology was consistent with a diagnosis of GSD IV: micronodular cirrhosis, PAS-positive diastase-resistant cytoplasmic inclusions, and amylopectin filaments on electron microscopy. A decision was made to proceed with liver transplantation. At 20 months of age, the patient successfully underwent the transplant procedure. Explant pathology noted micronodular cirrhosis and PAS-positive diastase-resistant cytoplasmic inclusions across the majority of the liver. There was mild to moderate chronic inflammatory infiltrate predominant within the portal tracts with lymphocytes, rare plasma cells, and rare eosinophils. Post-operatively, his course was uncomplicated with no documented episodes of acute cellular rejection. His last echocardiogram was done at 16.8 years and was normal. He has no known neuromuscular involvement. It remains unclear whether the patient has undergone genetic testing to confirm the suspected diagnosis; his last reported follow up was at 18.1 years and he has not been evaluated by a genetics specialist, and no genetic testing report was available in his medical record.

Supplementary Figure S1. Liver histology images from patients with hepatic glycogen storage disease type IV.

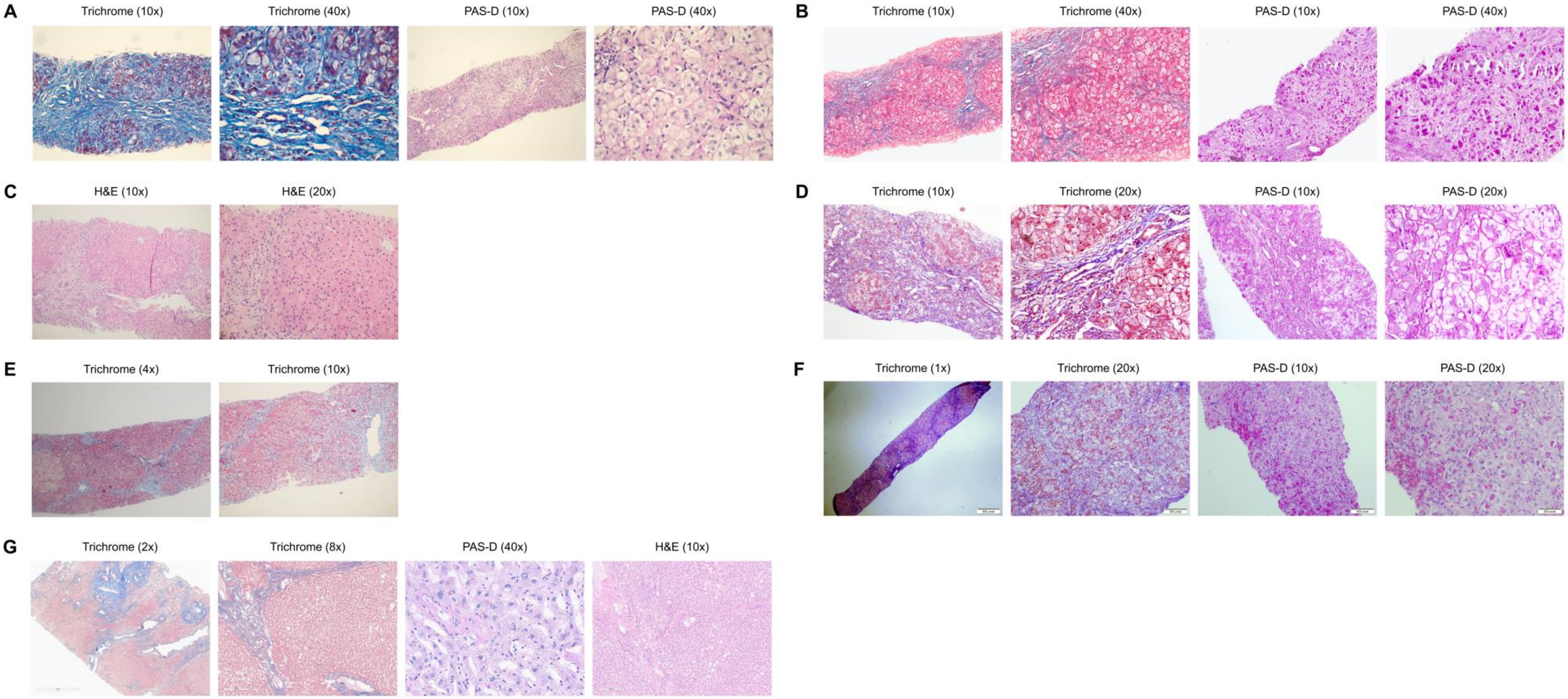

Representative images of available liver histology slides stained with Masson's trichrome ("Trichrome"), periodic acid-Schiff (PAS) with diastase treatment ("PAS-D"), and/or H&E at low and high magnification from patients with glycogen storage disease type IV. **(A)** Patient C1: Liver biopsy at 1.4 years of age demonstrated advanced cirrhosis with parenchymal extinction. **(B)** Patient C5: Liver biopsy at 1.0 years of age demonstrated advanced cirrhosis with parenchymal extinction. **(C)** Patient C12: Liver biopsy at 3.1 years of age demonstrated cirrhotic-like morphology with a 'biliary' pattern (extensive portal to portal bridging with preserved central veins). **(D)** Patient C16: Liver biopsy at 1.2 years of age demonstrated advanced cirrhosis with parenchymal extinction. **(E)** Patient C18: Liver biopsy at 4.6 years of age demonstrated abundant portal to portal bridging with focal equivocal nodule formation. **(F)** Patient C36: Liver biopsy at 1.1 years of age demonstrated advanced cirrhosis with parenchymal extinction. **(G)** Patient C45: Autopsy liver samples at 47.8 years demonstrated bridging fibrosis and cirrhosis.

Supplementary Figure S2. Quantitative measurement of liver fibrosis in *Gbe1*$^{ys/ys}$ mouse model.

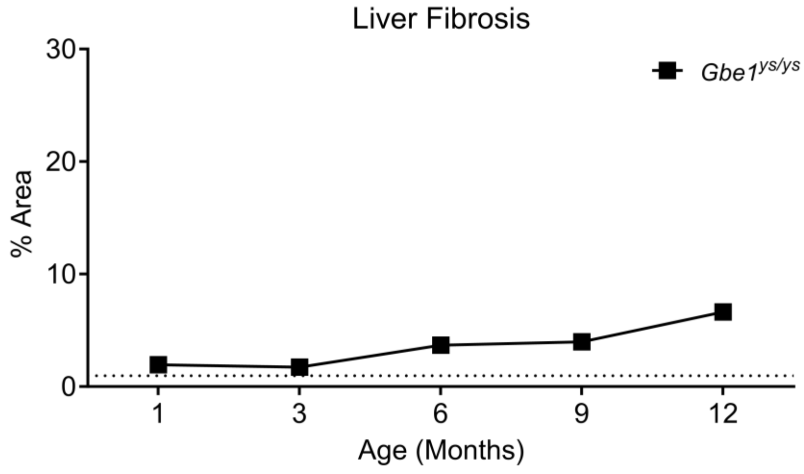

The extent of liver fibrosis was quantified in Masson's trichrome-stained liver histology slides. The area fraction (%) of fibrosis was measured in representative images of histology slides (3-6 images per mouse) from *Gbe1*$^{ys/ys}$ mice ages 3 to 12 months using a trainable Weka segmentation model in ImageJ. Data presented as mean. The dotted line represents WT levels at 6 months of age for comparison.
