# Supplementary material for: Natural history study of hepatic glycogen storage disease type IV and comparison to Gbe1ys/ys model
Source: JCI Insight. 2024 May 14;9(12):e177722. doi: 10.1172/jci.insight.177722 (PMC11383185; doi:10.1172/jci.insight.177722)
Supplement: Supplemental table 1 [file jciinsight-9-177722-s250.pdf]

Supplemental Table 1. Characteristics of patients with hepatic GSD IV

ID	Sex	Care location[A]	Race, ethnicity, ancestry	GBE activity (control)[B]	*GBE1* allele 1	*GBE1* allele 2	Biopsies[C]	Age at LT	Age at last follow-up[D]	Previously published[E]
C1	M	USA	American Indian, German, English	L: 7.5 μmol/min/g tissue (85 ± 31); F: 89 nmol/min/mg protein (1,300 ± 390)	c.691 + 2T > C [P]	c.1544G > A (p.R515H) [P/LP]	Liver: 1.4; Muscle: 1.6	1.6	12.1	NA
C3	F	USA	Ashkenazi Jewish	F: 172 nmol/min/mg protein (1,131)	c.986A > C (p.Y329S) [P]	c.671T > C (p.L224P) [P/LP]	Liver: 2.2	NA	29.7	Patient C (9); Patient 2 (12)
C4	M	USA	ND	H: 2.0 μmol/min/g tissue (> 2.6)	c.808C > T (p.Q270X) [P]	c.1544G > A (p.R515H) [P/LP]	Liver: 0.9; Heart: 1.1	1.2	1.7[D]	NA
C5	M	Canada	ND	ND	c.1241T > C (p.V414A) [NR]	c.1544G > A (p.R515H) [P/LP]	Liver: 1.0; Muscle: 1.2;	2.4	13.8	NA

							Heart: 1.2			
C6	M	USA	Polish, English, German	L: 0 μmol/min/g tissue (85 ± 31)	ND	ND	Liver: ND	0.9	13.8	NA
C7	M	USA	ND	F: 125 nmol/min/mg protein (1,131)	ND	ND	Liver: 4.1	NA	25.3	Patient D (9)
C8	M	USA	ND	ND	ND	ND	Liver: 2.5; Heart: 24.2	2.7	32.2	NA
C9	F	Germany	ND	E: 1.0 μmol/min/g Hb (8–25); W: 0.04 μmol/min/mg protein (0.15–0.5)	c.691 + 2T > C [P]	c.1883A > G (p.H628R) [P/LP/VUS]	ND	NA	6.2	Patient 10 (18)
C10	M	USA	Irish, American Indian	ND	c.691+2T > C [P]	c.1544G > A (p.R515H) [P/LP]	Liver: 1.7	NA	5.2	NA
C11[F]	M	USA	Israeli, Ashkenazi Jewish	ND	c.986A > C (p.Y329S) [P]	c.1108 + 5G > A	ND	NA	6.1	Patient 9 (18)

						(p.I500T) [VUS]				
C12[F]	M	USA	Israeli, Ashkenazi Jewish	ND	c.986A > C (p.Y329S) [P]	c.1108 + 5G > A (p.I500T) [VUS]	Liver: 3.1; Muscle: 8.3	NA	8.9	Patient 8 (18)
C13	M	Canada	Mennonite	ND	c.691 + 2T > C [P]	c.760A > G (p.T254A) [P/LP/VUS]	Liver: 2.7	NA	5.1	NA
C16	M	India	Asian	ND	c.476C > T (p.P159L) [P]	c.476C > T (p.P159L) [P]	Liver: 1.2	NA	2.0[D]	NA
C18	F	Nicaragua	Hispanic	L: 3.1 µmol/min/g tissue (> 5.4)	c.1621A > G (p.N541D) [P/LP]	c.1655C > T (p.P552L) [LP]	Liver: 4.6; Muscle: 4.6	NA	8.3	NA
C26[G]	M	USA	Hispanic	L: "deficient/complete absence"	c.1543C > T (p.R515C) [P/LP]	c.1484T > C (p.M495T) [VUS]	Liver: 2.0	2.3	19.4	NA
C32[G]	F	USA	Hispanic	F: 254 nmol/min/mg	c.1543C > T (p.R515C) [P/LP]	c.1484T > C	ND	3.5	13.9[D]	NA

				protein (1,300 ± 390)		(p.M495T) [VUS]				
C36	M	India	Asian	ND	c.476C > T (p.P159L) [P]	c.476C > T (p.P159L) [P]	Liver: 1.1	1.3	1.3	NA
C43	F	Brazil	ND	ND	ND	ND	Liver: 1.4	2.3	12.2	NA
C44	F	Brazil	ND	ND	c.480G > A (p.W160X) [P/LP]	c.1544G > A (p.R515H) [P/LP]	Liver: 0.8	1.2	1.4	NA
C45	M	USA	Native Hawaiian	F: 150–180 nmol/min/mg protein (1500 ± 450) and 163.5 nmol/min/mg protein (612–1850)	c.986A > C (p.Y329S) [P]	c.2003delA [NR]	Liver: 2.5; Liver: 47.8[H]	NA	47.8[D]	Patient B (9); the patient (10)
C61	M	USA	Middle Eastern	ND	c.998A > T (p.E333V) [P/LP]	c.292G > C (p.V98L) [VUS]	ND	3	19.3	NA
C63	M	USA	Hispanic	L: "deficient"	c.1468delC (p.L490Wfs*5) [NR]	c.1544G > A (p.R515H) [P/LP]	Liver: 0.3	NA	1.1[D]	NA

C66	M	USA	ND	L: “consistent with a GSD IV diagnosis”	ND	ND	Liver: 0.9	1.7	18.1	

All ages at biopsy, autopsy, LT, and last follow-up provided in years. Variants in *GBE1* correspond with accession NM_000158.4, and ClinVar interpretation as of September 2023 was included in brackets with P, pathogenic; LP, likely pathogenic; VUS, variant of uncertain significance; NR, not reported in ClinVar. [A]The patient’s primary care location (country) at the time of study enrollment. [B]L, liver; F, skin fibroblasts; H, heart; E, erythrocytes; W, white blood cells (leukocytes). Reported control GBE values are listed in parentheses and in the same units as the patient GBE values. Quotations used to describe interpretation by the reporting laboratory if specific values were not documented in the medical record. [C]Patient ages at any available liver, muscle, or heart biopsies obtained prior to liver transplantation (LT), if applicable, are reported. Other biopsies (e.g., post-LT liver biopsies or gastrointestinal biopsies from esophagogastroduodenoscopy) are reported in Supplemental Data 1. [D]For deceased patients (denoted with “D”), the age at death is reported as the age at last follow-up. [E]For patients with clinical data previously published, the reference and identifier are provided. [F]Sibling pair. [G]Sibling pair. [H]Collected during autopsy. NA, not applicable; ND, no data/not assessed.
